# Long-term safety and exploratory efficacy of fevipiprant in patients with inadequately controlled asthma: the SPIRIT randomised clinical trial

**DOI:** 10.1186/s12931-021-01904-8

**Published:** 2021-12-11

**Authors:** Jorge Maspero, Ioana Octavia Agache, Tadashi Kamei, Makoto Yoshida, Bryan Boone, James M. Felser, Fernando Kawakami, Barbara Knorr, David Lawrence, Thomas Lehmann, Wei Wang, Andrew J. Pedinoff

**Affiliations:** 1 Allergy and Respiratory Research Unit, Fundación CIDEA, Buenos Aires, Argentina; 2 Transylvania University, Brasov, Romania; 3 Kamei Internal Medicine and Respiratory Clinic, Takamatsu-city, Kagawa Japan; 4 National Hospital Organization Fukuoka National Hospital, Fukuoka-city, Fukuoka Japan; 5 Novartis Pharmaceuticals Corporation, East Hanover, NJ USA; 6 Novartis Pharma AG, Basel, Switzerland; 7 Novartis Institutes for Biomedical Research Co., Ltd, Shanghai, China; 8 Princeton Center for Clinical Research, Skillman, NJ USA

**Keywords:** Adverse event, DP_2_ receptor, Fevipiprant, Safety, Uncontrolled asthma

## Abstract

**Background:**

The prostaglandin D_2_ (PGD_2_) receptor 2 (DP_2_ receptor) pathway is an important regulator of the inflammatory cascade in asthma, which can be stimulated by allergic or non-allergic triggers. Fevipiprant is an oral, non-steroidal, highly selective, reversible antagonist of the DP_2_ receptor that inhibits the binding of PGD_2_ and its metabolites.

**Methods:**

SPIRIT, a 2-treatment period (52-week, double-blind and optional 104-week single-blind), randomised, placebo-controlled, multicentre, parallel-group study, assessed the long-term safety of fevipiprant (150 mg and 450 mg o.d.) added to standard of care in patients ≥ 12 years with uncontrolled asthma. Stratified block randomisation was used. Patients were randomised in an approximate ratio of 3:3:1 (fevipiprant 150 mg, fevipiprant 450 mg or placebo). Patients were either newly enrolled or had participated in a previous fevipiprant Phase 3 trial. Primary endpoints were: time-to-first treatment emergent adverse event (AE); serious AE; and AE leading to discontinuation from study treatment. Data from both treatment periods were combined for analyses. Data were collected during study site visits.

**Results:**

In total, 1093 patients were randomised to receive fevipiprant 150 mg, 1085 to fevipiprant 450 mg, and 360 to placebo. Overall, 1184 patients had ≥ 52 weeks’ treatment, while 163 received ≥ 104 weeks’ treatment. Both doses were well tolerated, with a safety profile similar to placebo both in new patients and in those enrolled from previous studies.

In exploratory analyses, reduced rates of moderate-to-severe asthma exacerbations, increased time-to-first moderate-to-severe asthma exacerbation and improved FEV_1_ were observed for both doses of fevipiprant versus placebo; these were without multiplicity adjustment and should be interpreted with caution. SPIRIT was terminated early, on 16 December 2019, by the Sponsor.

**Conclusions:**

In patients with uncontrolled asthma, the addition of fevipiprant had a favourable long-term safety profile.

***Trial registration*:**

Clinicaltrials.gov, NCT03052517, prospectively registered 23 January 2017, https://clinicaltrials.gov/ct2/show/NCT03052517.

**Supplementary Information:**

The online version contains supplementary material available at 10.1186/s12931-021-01904-8.

## Background

Asthma is the most prevalent chronic respiratory disease, affecting over 350 million people worldwide currently [1]; this is estimated to increase to over 400 million by 2025 [2]. Although approximately 24% of adults with asthma receive therapy according to Step 4 or 5 of the Global Initiative for Asthma (GINA) strategy, many of these patients have poor symptom control [3]. Therefore, new therapeutic options for patients not achieving adequate asthma control are needed.

The prostaglandin D_2_ receptor 2 (DP_2_ receptor) is expressed on a broad range of key inflammatory and structural cells involved in the pathogenesis of asthma. It is a chemokine receptor with a diverse range of ligands in addition to prostaglandin D_2_ (PGD_2_), including several PGD_2_‐derived metabolites [4].

Fevipiprant is an oral, non-steroidal, highly selective, reversible antagonist of the DP_2_ receptor that prevents the binding of PGD_2_ [5] and its metabolites to the DP_2_ receptor. Binding of fevipiprant to the DP_2_ receptor reduces the migration and activation of eosinophils, basophils, innate lymphoid cells (ILC-2) and T lymphocytes into the airway tissues and reduces the PGD_2_-driven release of Th2 cytokines [6, 7]. Post hoc subgroup analysis of a Phase 2 proof of concept study, in patients with reduced lung function at baseline, showed that fevipiprant 500 mg once daily significantly improved pre-dose trough forced expiratory volume in one second (FEV_1_) and asthma control (assessed with the Asthma Control Questionnaire [ACQ] scores) compared with placebo [8] despite the absence of significant improvements in these measurements across the overall population. Other Phase 2 studies showed that optimal dose response was achieved with fevipiprant 150 mg once daily [9] and that patients treated with fevipiprant 225 mg twice daily had a 3.5-times greater reduction in sputum eosinophils compared with placebo, although a significant effect on blood eosinophil count was not demonstrated [10].

In the ZEAL-1 (NCT03215758) and ZEAL-2 (NCT03226392) replicate Phase 3 studies, in patients with moderate uncontrolled asthma (GINA steps 3 and 4) receiving 150 mg fevipiprant once daily, no significant improvements were observed in lung function or in other asthma related outcomes, such as daytime symptom score or quality of life (AQLQ + 12 score) [11]. Similarly, in two replicate Phase 3 pivotal trials, LUSTER-1 (NCT02555683) and LUSTER-2 (NCT02563067), which investigated the effect of two doses of fevipiprant on the reduction of exacerbations in patients with severe asthma, no significant effect of fevipiprant was shown in patients with severe asthma (GINA steps 4 and 5) treated with the 450 mg dose of fevipiprant once daily [12]. Despite promising results in Phase II and some suggestion of efficacy in the LUSTER trials, the findings of the Phase III clinical trial programme of fevipiprant indicated that the molecule was not sufficiently efficacious to continue its clinical development.

The SPIRIT study (NCT03052517) was a two-treatment period, randomised, placebo-controlled, multicentre parallel-group study enrolling patients who rolled over from either the LUSTER or the ZEAL studies, as well as patients who had not previously been part of a fevipiprant study. The primary objective of this study was to assess the long-term safety of fevipiprant when added to existing standard of care (SoC) asthma therapy in patients with uncontrolled asthma at GINA steps 3, 4 or 5. The study also included an exploratory efficacy analysis of fevipiprant assessed by exacerbation rate and lung function. The safety and exploratory efficacy results of this study are presented here.

## Materials and methods

### Trial design

**Fig. 1:**
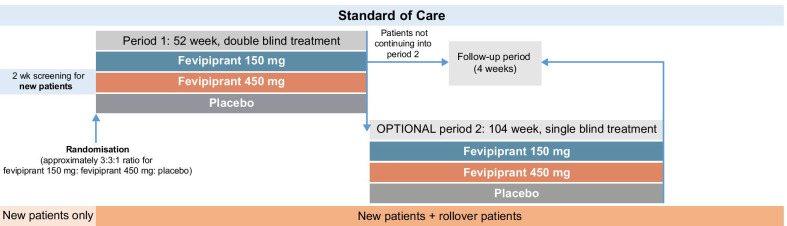
SPIRIT study design. Patients aged 12 years and older with inadequately controlled moderate-to-severe asthma receiving Global Initiative for Asthma (GINA) Steps 3, 4 and 5 standard of care asthma therapy were randomly assigned (3:3:1) to receive either fevipiprant 150 mg, fevipiprant 450 mg, or placebo once daily

SPIRIT was a two-treatment period, multicentre, placebo-controlled Phase 3 study: the first period was a 52-week, double-blind study followed by an optional 104-week, single-blind second period (Fig. 1). The study was terminated early by the Sponsor when the overall data from Phase 3 trials (LUSTER [12] and ZEAL [11]) did not support further development of fevipiprant. The initial intention was to analyse Treatment Period 1 separately but due to the study’s early termination, data from the two treatment periods were combined for the study analyses. An independent data monitoring committee oversaw patient safety data for the study.

### Patients

The study population included male or female patients ≥ 12 years of age with moderate-to-severe asthma (GINA steps 3, 4, and 5) who were receiving asthma treatment according to GINA guidelines. This study included two cohorts of patients: rollover patients who had completed any of the four Phase 3 pivotal fevipiprant efficacy studies on active study treatment (LUSTER-1, LUSTER-2, ZEAL-1 or ZEAL-2) and new patients who had not previously participated in a fevipiprant study.

Inclusion criteria for previous Phase 3 studies have been published [11, 12]. Patients who had participated in a prior Phase 3 fevipiprant study were excluded if they did not complete the prior study on blinded therapy or had a serious and drug-related adverse event (AE) during the prior study. New patients were male or female aged ≥ 12 years with inadequately controlled asthma on treatment at GINA steps 4 and 5 for at least 3 months prior to screening and an ACQ score ≥ 1.5 at inclusion. The required cut-off for the % predicted FEV_1_ for new patients was ≤ 85% for patients aged ≥ 18 years, and ≤ 90% for patients aged 12 to < 18 years. New patients were excluded if they had an asthma exacerbation requiring systemic corticosteroids, hospitalisation, or an emergency room visit within six weeks, or if they had a respiratory tract infection or asthma worsening within four weeks of the first visit. See Additional file 1 for full inclusion and exclusion criteria.

### Study treatment

Patients were randomised to receive either fevipiprant 150 mg, fevipiprant 450 mg, or placebo. The overall approximate randomisation ratio was 3:3:1 (fevipiprant 150 mg: fevipiprant 450 mg: placebo). Rollover patients were re-randomised to treatment and may have received a different treatment than that received in their prior Phase 3 study. Further information on randomisation is found in the Additional file 1.

### Study endpoints

The primary endpoint was to evaluate the long-term safety of fevipiprant (150 and 450 mg o.d.) versus placebo by assessing: treatment emergent AEs; treatment emergent serious adverse events (SAEs); and study treatment discontinuations due to treatment emergent AEs. AEs (including asthma exacerbations), starting on or after the time of the first intake of study drug and until the day after the last intake of study drug were classified as treatment emergent.

In addition, the study included exploratory efficacy analysis of fevipiprant (150 mg o.d. and 450 mg o.d.) versus placebo assessed by the rate of moderate-to-severe asthma exacerbations and the average change from baseline in pre-dose FEV_1_. A severe asthma exacerbation was defined as treatment with ‘rescue’ systemic corticosteroids for ≥ 3 days and hospitalisation; or treatment with ‘rescue’ systemic corticosteroids for ≥ 3 days and emergency department visit (> 24 h); or death due to asthma. A moderate asthma exacerbation was defined as treatment with ‘rescue’ systemic corticosteroids for ≥ 3 days either as an outpatient or in emergency department visits (≤ 24 h). For rollover patients, written informed consent for Treatment Period 1 (and assent, if applicable) was obtained prior to randomisation. For new patients entering Treatment Period 1, informed consent (and assent, if applicable) was obtained within 14 days prior to or at Screening and before any study-related assessments or procedures were performed. For Treatment Period 2, written informed consent (and assent, if applicable) was obtained at Visit 301 (first visit of Treatment Period 2) for patients who agreed to participate in this optional 104-week treatment period.

### Statistical analysis

#### Analysed sets

Safety endpoints were analysed for all patients who received at least one dose of study drug during SPIRIT. The analysis included patients who rolled over from LUSTER-1, LUSTER-2, ZEAL-1 and ZEAL-2 fevipiprant trials in addition to newly randomised patients.

The study initially planned to include all patients in the exploratory efficacy analysis. However, due to the negative results on FEV_1_ for the ZEAL trials and the assumption that exacerbations would be rare in this patient population of moderate asthma severity, prior to the SPIRIT database lock the decision was made to exclude the ZEAL patients from the exploratory efficacy analysis. Therefore, exploratory analyses of efficacy were performed on the pool of randomised patients who either rolled over from LUSTER-1 or LUSTER-2 or were newly randomised. Patients were analysed according to treatment received.

#### Safety analyses

The primary safety variables were time-to-first treatment emergent AE, SAE, and AE leading to discontinuation from study treatment. The primary variable was analysed using a Cox regression model stratified by randomisation stratum, with treatment group, severity of asthma and region as fixed factors. Randomisation strata were as follows: patients on fevipiprant 150 mg once daily treatment in LUSTER-1 and LUSTER-2; patients on fevipiprant 450 mg once daily treatment in LUSTER-1 and LUSTER-2; patients on placebo in LUSTER-1 and LUSTER-2; patients on fevipiprant 150 mg once daily treatment in ZEAL-1 and ZEAL-2; patients on placebo in ZEAL-1 and ZEAL-2, and patients who had not previously participated in a study of fevipiprant. Hazard ratios were calculated comparing fevipiprant 450 or 150 mg to placebo for time-to-first treatment-emergent AE/SAE/AE leading to treatment discontinuation.

All AEs that occurred after patients received first dose of treatment were included in the analysis. AEs were coded using MedDRA version 22.1. Descriptive summary statistics for laboratory parameters, vital signs, and ECG parameters were provided.

#### Efficacy analyses

All efficacy analyses were exploratory and were performed on the pool of randomised patients who either rolled over from LUSTER-1/LUSTER-2 or were newly randomised. The rate of on-treatment moderate-to-severe asthma exacerbations was analysed by a negative binomial regression model. The model included randomisation stratum, treatment, severity of asthma (GINA steps 4, and 5), region and the natural logarithm of the number of asthma exacerbations in the 12 months prior to screening. For the overall population, blood eosinophil count at screening (≥ 250 cells/µL, < 250 cells/µL) was also included as a covariate, aligning with the analysis in the LUSTER study [12]. Sputum eosinophil levels were not measured. For rollover patients, asthma exacerbation rate and blood eosinophil count recorded at screening of the previous study are used. The log (duration of follow-up in years) is used as an off-set variable.

Change from baseline in pre-dose FEV_1_ was analysed using a mixed model for repeated measures with an unstructured covariance structure. The model included the same covariates as used for the analysis of exacerbations but replaced the number of asthma exacerbations in the 12 months prior to screening with baseline pre-dose FEV_1_. Interaction terms for baseline FEV_1_ by visit and treatment by visit were also included. To further investigate the subgroup of patients with high eosinophils, the efficacy analyses for moderate-to-severe asthma exacerbations and pre-dose FEV_1_ were performed for patients with eosinophil count ≥ 250 cells/μL (high eosinophils subpopulation) at Visit 1 and the overall study population in relevant models. For rollover patients, blood eosinophil count at Visit 1 of the previous study was considered.

## Results

### Patient disposition

In total, 2538 patients were enrolled/randomised in the study (1807 rollover plus 731 new patients). The number of randomised patients per study group was 1093 for fevipiprant 150 mg, 1085 for fevipiprant 450 mg, and 360 for placebo (Table 1). The study was initiated on 21 March 2017 and was terminated early on 16 December 2019 (Additional file 1: Fig. S1).

**Table 1:** Baseline characteristics

	Fevipiprant 150 mg N = 1092	Fevipiprant 450 mg N = 1084	Placebo N = 361	Total N = 2537
Demographic characteristics				
Age, years, mean (SD)	50.1 (14.95)	50.1 (15.55)	49.9 (14.99)	50.1 (15.21)
Female sex, n (%)	659 (60.3)	666 (61.4)	229 (63.4)	1554 (61.3)
Disease characteristics				
Duration of asthma, years, n	1090	1084	361	2535
Mean (SD)	21.01 (14.974)	21.03 (15.243)	19.75 (14.023)	20.84 (14.960)
Number of asthma exacerbations in the previous year, n	1092	1084	361	2357
Mean (SD)	1.39 (1.315)	1.41 (1.264)	1.45 (1.512)	1.41 (1.323)
Atopic status—n (%)				
Yes	670 (61.4)	632 (58.3)	218 (60.4)	1520 (59.9)
No	418 (38.3)	450 (41.5)	142 (39.3)	1010 (39.8)
Missing	4 (0.4)	2 (0.2)	1 (0.3)	7 (0.3)
Prior participation in a fevipiprant phase 3 study, n (%)				
LUSTER-1/LUSTER-2	449 (41.1)	442 (40.8)	149 (41.3)	1040 (41.0)
150 mg QD	254 (23.3)	53 (4.9)	51 (14.1)	358 (14.1)
450 mg QD	48 (4.4)	241 (22.2)	47 (13.0)	336 (13.2)
Placebo	148 (13.6)	148 (13.7)	50 (13.9)	346 (13.6)
ZEAL-1/ZEAL-2	330 (30.2)	329 (30.4)	107 (29.6)	766 (30.2)
150 mg QD	218 (20.0)	112 (10.3)	53 (14.7)	383 (15.1)
Placebo	112 (10.3)	217 (20.0)	54 (15.0)	383 (15.1)
Newly randomised	313 (28.7)	313 (28.9)	105 (29.1)	731 (28.8)
Smoking history—n (%)				
Never	892 (81.7)	906 (83.6)	298 (82.5)	2096 (82.6)
Former	200 (18.3)	178 (16.4)	63 (17.5)	441 (17.4)
Blood eosinophil at screening—n (%)				
˂250 cells/μL	434 (39.7)	427 (39.4)	149 (41.3)	1010 (39.8)
≥ 250 cells/μL	635 (58.2)	641 (59.1)	202 (56.0)	1478 (58.3)
Missing	23 (2.1)	16 (1.5)	10 (2.8)	49 (1.9)
Baseline spirometry				
Percent predicted FEV_1_ (%) (Pre-bronchodilator)				
n	1079	1069	354	2502
Mean	61.0	60.5	60.9	60.8
SD	13.86	13.98	14.37	13.98
FEV_1_ reversibility (%)				
n	1069	1064	353	2486
Mean	21.9	22.9	24.0	22.6
SD	17.97	18.82	16.56	18.16

Age is calculated from date of screening and July 1st of the year of birth for adults. For adolescents, age is collected directly from Electronic Case Report Form (eCRF)

For all other variables, prior study baseline is used as the baseline value in patients who completed a prior Phase 3 study, and SPIRIT baseline is used as the baseline value in new patients

Duration of asthma is calculated as date of asthma first diagnosed until Visit 1. Blood eosinophil count at screening visit of prior study is considered for rollover patients. FEV_1_ reversibility is calculated as increase of FEV_1_ value after inhalation of bronchodilator relative to the FEV_1_ value before inhalation of bronchodilator. Reversible: increase of FEV_1_ value ≥ 12% and ≥ 200 mL Not reversible: change of FEV_1_ value < 12% or < 200 mL. FEV_1_ reversibility demonstrated at clinic at Visit 1 are presented. Percent predicted FEV_1_: percentage of FEV_1_ relative to the predicted normal value. For patients who completed a prior Phase 3 study, prior study baseline is used as the baseline value

*BMI* body mass index; *FEV*_1_ forced expiratory volume in one second; *SD* standard deviation

### Baseline demographics

Demographic characteristics were well balanced across treatment groups (Table 1). The treatment groups were also balanced in terms of baseline disease characteristics, including duration of asthma (mean duration 20.8 years), atopic status (59.9% atopic), and smoking status (82.6% never smoked, 17.4% former smokers).

Lung function, based on spirometry assessments, was comparable across the treatment groups (Table 1). The mean predicted pre-bronchodilator FEV_1_ was 60.8% (measured after withholding bronchodilator at screening). The mean percentage increase in FEV_1_ after bronchodilator inhalation (FEV_1_ reversibility) was 22.6%.

### Exposure to fevipiprant

At study termination, 1184 patients had received at least 52 weeks of treatment in SPIRIT and 163 patients had received at least 104 weeks of treatment (Additional file 1: Table S1).

### Safety

The hazard ratios were < 1 for time-to-first treatment-emergent AE and for time-to-first treatment-emergent SAE for both fevipiprant treatment groups compared with placebo (Table 2). The hazard ratio was > 1 for time-to-first treatment-emergent AE leading to treatment discontinuation for fevipiprant treatment groups compared with placebo. However, the number of events was relatively small in all treatment arms and the confidence intervals around the estimates are wide (Table 2).

**Table 2:** Time-to-first treatment-emergent AE, SAE and AE leading to study treatment discontinuation

Treatment	n (%)	Comparison	Hazard ratio	95% CI
Time to 1st TEAE				
Fevipiprant 150 mg (m = 1081)	709 (65.6)	fevipiprant 150 mg/placebo	0.88	(0.76, 1.02)
Fevipiprant 450 mg (m = 1077)	681 (63.2)	fevipiprant 450 mg/placebo	0.85	(0.73, 0.99)
Placebo (m = 359)	243 (67.7)	fevipiprant 450 mg/fevipiprant 150 mg	0.97	(0.86, 1.08)
Time to 1st treatment emergent SAE				
Fevipiprant 150 mg (m = 1081)	86 (8.0)	fevipiprant 150 mg/placebo	0.80	(0.54, 1.22)
Fevipiprant 450 mg (m = 1077)	63 (5.8)	fevipiprant 450 mg/placebo	0.63	(0.41, 0.97)
Placebo (m = 359)	33 (9.2)	fevipiprant 450 mg/fevipiprant 150 mg	0.78	(0.55, 1.11)
Time to 1st TEAE leading to treatment discontinuation				
Fevipiprant 150 mg (m = 1081)	30 (2.8)	fevipiprant 150 mg/placebo	1.14	(0.56, 2.56)
Fevipiprant 450 mg (m = 1077)	37 (3.4)	fevipiprant 450 mg/placebo	1.33	(0.67, 2.95)
Placebo (m = 359)	9 (2.5)	fevipiprant 450 mg/fevipiprant 150 mg	1.17	(0.70, 1.96)

The Cox regression model = treatment group + severity of asthma (GINA treatment steps 3, 4 and 5) + region as fixed class effects, stratified by randomization stratum (fevipiprant 150 mg once daily in LUSTER-1/LUSTER-2, fevipiprant 450 mg once daily in LUSTER-1/LUSTER-2, Placebo in LUSTER-1/LUSTER-2, fevipiprant150 mg once daily in ZEAL-1/ZEAL-2, Placebo in ZEAL-1/ZEAL-2, New patients). Patients without the event of interest were censored at the minimum out of the dates of last medication intake + 30 days, final visit date, and date of death

A hazard ratio < 1 favors the treatment group in the numerator of the ratio

*AE* adverse event; *n* number of patients with at least one event; *m* total number of patients included in the analysis; *TEAE* treatment emergent AE

Both doses of fevipiprant were well tolerated, with a safety profile similar to that of placebo in terms of type, severity, and frequency of AEs (Table 3). The safety profile was comparable between new patients and patients rolled over from previous Phase 3 studies (Table 4). There was no observed effect on the overall safety profile resulting from the different exposures to treatment in the patients who were newly recruited compared with those sourced from previous Phase 3 studies. In addition, there was no observed effect on AEs of age, sex or age of onset of disease (Additional file 1: Tables S2–S4).

**Table 3:** Overall summary of exposure adjusted incidence rates of treatment emergent adverse events

	Fevipiprant 150 mg (N = 1092) Exp. = 1100.8 PY n IR	Fevipiprant 450 mg (N = 1084) Exp. = 1080.2 PY n IR	Placebo (N = 361) Exp. = 364.1 PY n IR
Patients with AE(s)	716 65.0	686 63.5	245 67.3
SAE(s)	87 7.9	64 5.9	33 9.1
SAE(s) with an outcome of death	3 0.3	1 0.1	1 0.3
Discontinued study treatment due to any AE(s)	30 2.7	37 3.4	9 2.5
Discontinued study treatment due to any SAE(s)	15 1.4	13 1.2	3 0.8

IR (incidence rate per 100 patient years) = n/(sum of patient exposure) × 100

A patient with multiple AEs is counted only once in the AE category for that treatment

*AE* adverse event; *n* number of patients with events; *PY* person years; *SAE* serious adverse event

**Table 4:** Overall summary of exposure adjusted incidence rates of treatment emergent adverse events by source of patients

Completed LUSTER-1/LUSTER-2			
	Fevipiprant 150 mg N = 449 Exp. = 513.7 PY n IR	Fevipiprant 450 mg N = 442 Exp. = 501.6 PY n IR	Placebo N = 149 Exp. = 174.5 PY n IR
Patients with AE(s)	323 62.9	289 57.6	112 64.2
SAE(s)	35 6.8	27 5.4	18 10.3
SAE(s) with an outcome of death	1 0.2	0 0	1 0.6
Discontinued study treatment due to any AE(s)	13 2.5	13 2.6	4 2.3
Discontinued study treatment due to any SAE(s)	7 1.4	5 1.0	0 0

Completed ZEAL-1/ZEAL-2			
	Fevipiprant 150 mg N = 330 Exp. = 222.5 PY n IR	Fevipiprant 450 mg N = 329 Exp. = 218.2 PY n IR	Placebo N = 107 Exp. = 72.5 PY n IR
Patients with AE(s)	177 79.6	179 82.0	61 84.1
SAE(s)	12 5.4	7 3.2	6 8.3
SAE(s) with an outcome of death	1 0.4	0 0	0 0
Discontinued study treatment due to any AE(s)	8 3.6	8 3.7	2 2.8
Discontinued study treatment due to any AE(s)	1 0.4	1 0.5	1 1.4

New patients			
	Fevipiprant 150 mg N = 313 Exp. = 364.6 PY n IR	Fevipiprant 450 mg N = 313 Exp. = 360.3 PY n IR	Placebo N = 105 Exp. = 117.1 PY n IR
Patients with AE(s)	216 59.2	218 60.5	72 61.5
SAE(s)	40 11.0	30 8.3	9 7.7
SAE(s) with an outcome of death	1 0.3	1 0.3	0 0
Discontinued study treatment due to any AE(s)	9 2.5	16 4.4	3 2.6
Discontinued study treatment due to any AE(s)	7 1.9	7 1.9	2 1.7

A patient with multiple AEs is counted only once in the AE category for that treatment

*AE* adverse event; *n* number of patients with events; IR (incidence rate per 100 patient years) = n/(sum of patient exposure) × 100; *PY* person years; *SAE* serious adverse event

Asthma exacerbation, nasopharyngitis and bronchitis were the most frequent AEs reported across the treatment groups (Additional file 1: Table S5). All other AEs are reported in exposure-adjusted incidence rate (EAIR) < 10. Most of the events were mild or moderate in intensity (Additional file 1: Table S6). Asthma exacerbations were the most frequent SAE (Additional file 1: Table S7) with EAIRs of 2.7, 1.5 and 3.6 for patients treated with fevipiprant 150 mg, fevipiprant 450 mg and placebo, respectively. Few patients had AEs that led to discontinuation (Additional file 1: Table S8) (EAIR: 2.7 in the fevipiprant 150 mg group, 3.4 in the fevipiprant 450 mg group and 2.5 in the placebo group).

There were five treatment-emergent deaths reported in the study (three patients in the fevipiprant 150 mg group, one patient in the fevipiprant 450 mg group and one patient in the placebo group; associated SAEs are listed in the Additional file 1). There were no patient deaths due to treatment emergent asthma exacerbations. None of the deaths were suspected to be related to study drug by the Investigator. The death of the patient in the fevipiprant 450 mg group occurred 36 days after the patient was discontinued from the study (Day 352) due to an adverse event (severe colloid brain cyst). Most patients did not have asthma exacerbations requiring hospitalisations (≥ 93.3% in any treatment group). One intubation was reported in the placebo group; none were reported in the fevipiprant dose groups.

The analysis of adverse events of special interest (AESI) for the fevipiprant programme (cardiac AEs, hepatotoxicity, idiosyncratic drug reactions, and tachycardia) did not identify any safety concerns. Overall, AESI were reported with low incidences and with comparable incidences across study groups (EAIR: 4.2 in fevipiprant 150 mg, 5.6 in fevipiprant 450 mg, 6.6 in placebo). The majority of the reported AESI were mild or moderate. See Additional file 1 for further details.

Haematology, biochemistry, vital signs, and ECG results were comparable across all treatment groups (Additional file 1: Tables S9–S12).

### Exploratory efficacy

For both the overall population and the high eosinophils subpopulation, reductions were observed in the rates of moderate-to-severe asthma exacerbations for fevipiprant treatment groups versus placebo (Table 5, Additional file 1: Fig. S2). In the high eosinophils subpopulation, the time-to-first moderate-to-severe asthma exacerbation was increased by both fevipiprant doses as compared with placebo, with no difference between doses (Additional file 1: Fig. S3).

**Table 5:** On-treatment analysis of rate of moderate-to-severe asthma exacerbations during the total treatment period

Treatment	Annualized rate (95% CI)	Comparison	Rate ratio	95% CI
Overall population				
Fevipiprant 150 mg (n = 748)	0.4 (0.3, 0.4)	Fevipiprant 150 mg/Placebo	0.58	(0.44, 0.77)
Fevipiprant 450 mg (n = 744)	0.3 (0.3, 0.4)	Fevipiprant 450 mg/Placebo	0.55	(0.41, 0.74)
		Fevipiprant 450 mg/Fevipiprant 150 mg	0.95	(0.75, 1.20)
Placebo (n = 245)	0.6 (0.5, 0.8)			
Patients with blood eosinophil count ≥ 250 cells/μL				
Fevipiprant 150 mg (n = 457)	0.4 (0.3, 0.5)	Fevipiprant 150 mg /Placebo	0.61	(0.43, 0.87)
Fevipiprant 450 mg (n = 460)	0.4 (0.4, 0.5)	Fevipiprant 450 mg/Placebo	0.64	(0.44, 0.92)
		Fevipiprant 450 mg/Fevipiprant 150 mg	1.04	(0.78, 1.40)
Placebo (n = 145)	0.7 (0.5, 0.9)			

Negative binomial regression model: log (exacerbation rate) = randomization stratum

(fevipiprant 150 mg in LUSTER-1/LUSTER-2, fevipiprant 450 mg in LUSTER-1/LUSTER-2, Placebo in LUSTER-1/LUSTER-2, New patients) + treatment + severity of asthma (GINA steps 3, 4, and 5) + region + the natural logarithm of the number of asthma exacerbations in the 12 months prior to screening, and for overall population, plus blood eosinophil count at screening (≥ 250 cells/μL, < 250 cells/μL). For rollover patients, asthma exacerbation and blood eosinophil count recorded at screening of prior study are used. The log (duration of follow-up in years) is used as an off-set variable

A rate ratio < 1 favors treatment group in the numerator of the ratio

In rollover patients the rate of moderate-to-severe asthma exacerbations was decreased for both doses of fevipiprant compared with placebo in both the overall population and the high eosinophils subpopulation (Additional file 1: Table S13). This effect was not observed in newly enrolled patients (Additional file 1: Table S14). There was no difference between the fevipiprant dose groups.

Patients who rolled over from the LUSTER-1/LUSTER-2 studies had lower rates of moderate-to-severe asthma exacerbations, compared with the rates for patients who withdrew from the treatment, irrespective of fevipiprant dose (Additional file 1: Table S15). This was true both for patients who had received fevipiprant in the LUSTER studies and those who had received placebo. Fevipiprant also nominally reduced exacerbation rates compared with placebo for patients who had at least one exacerbation each year in two consecutive years before joining SPIRIT (Additional file 1: Table S16).

The least squares (LS) mean treatment difference in pre-dose FEV_1_ indicated a modest improvement for the two fevipiprant treatment doses over placebo at Week 52 (Additional file 1: Table 17). This improvement was more pronounced in the high eosinophilic subgroup (Additional file 1: Table 17).

## Discussion

Fevipiprant has demonstrated a favourable safety profile across previous Phase 2 and 3 studies and was well tolerated in over 2830 healthy volunteers and patients with asthma, allergic rhinitis, and atopic dermatitis at daily doses of 50–500 mg [8–10, 13]. In the present study both doses of fevipiprant were well tolerated, with a long-term safety profile generally similar to placebo. The overall safety profile was comparable for patients who were newly recruited or rolled-over from previous Phase 3 studies and was comparable with that observed in previous Phase 2 and 3 trials of fevipiprant [9, 11, 12]. These safety findings are generally consistent with the favourable safety profile observed with other DP_2_ receptor antagonists [14–18]. To date, there has been no indication of any ethnicity-, age-, or sex-specific safety signals for fevipiprant [8–10]. No relationship between specific safety parameters and fevipiprant doses has been observed previously [8–10]. Previous studies with other DP_2_ receptor antagonists have reported safety findings not seen with fevipiprant [19], e.g. it has been reported that nasopharyngitis is a more common adverse event in patients treated with a DP_2_ receptor antagonist compared with placebo [15], this was not the case in the SPIRIT study.

In the LUSTER studies, reduction of asthma exacerbations was observed compared with placebo, although this was not statistically significant [12]. The exploratory analysis in the current study showed an overall nominal reduction in the rates of moderate-to-severe asthma exacerbations and increase in time-to-first moderate-to-severe asthma exacerbation for fevipiprant treatment groups versus placebo for both the overall population and in the high eosinophils subpopulation. The magnitude of efficacy was greater in the SPIRIT study, i.e. the reduction in exacerbations was 42–52% in SPIRIT, compared with 23% in the LUSTER studies. Previous studies have indicated that increased blood eosinophil count may be associated with more frequent asthma exacerbations [20, 21]. In the present study the reduction in exacerbations was observed for both the overall population and the high eosinophil subpopulation. It was believed that fevipiprant would be effective in patients with higher eosinophil counts because of the observed reduction in sputum eosinophils in a Phase 2 study [10]. However, it is unlikely that all exacerbations are eosinophilic, and the rate of reduction in exacerbations in this study was similar in patients with elevated eosinophil counts and in patients overall. Considering that prostaglandin D_2_ has broad chemoattractant activity, this result suggests that prostaglandin D_2_ may have also affected the activity in the non-eosinophilic pathway [22].

The decrease in exacerbations was driven by the patients who rolled-over from LUSTER-1/LUSTER-2 and not by those newly enrolled. Numerous factors may have contributed to this including positive selection bias for patients completing the LUSTER studies; new patients having a lower number of exacerbations prior to randomisation compared with LUSTER; flexibility in background treatment (investigators were permitted to adjust an individual patient’s SoC in SPIRIT but not in LUSTER) and low variability in treatment for rollover patients who continued to receive fevipiprant (52 weeks LUSTER «run-in» on a stable asthma background medication). Additionally, there was potentially longer treatment duration for patients who participated in SPIRIT having previously completed one of the LUSTER studies and were randomised to receive fevipiprant in both studies. However, it is also possible that rollover patients may have shown the Hawthorne effect, whereby changes in an individual’s behaviour result from their awareness of being observed [23, 24], contributing to an overall increase in adherence and efficacy over the long time period of SPIRIT.

Phase 2 studies had indicated that fevipiprant is associated with improvement in lung function, with 500 mg o.d. significantly improving trough FEV_1_ in patients with baseline FEV_1_ < 70% compared with placebo [8]. In another study, both the 150 mg and 300 mg o.d. doses were associated with a significant improvement in pre-dose FEV_1_ when added to low-dose inhaled corticosteroids compared with placebo.[9] Although lung function was not improved by fevipiprant in the ZEAL-1/ZEAL-2 studies [11], some improvements were seen in the LUSTER studies, particularly for post-bronchodilator FEV_1_ [12]. In the exploratory analysis in SPIRIT, improvements in pre-dose FEV_1_ were seen for the two fevipiprant treatment doses over placebo at Week 52 for both the overall population and the high eosinophils subpopulation.

## Conclusions

The results of the Phase 3 clinical trial programme of fevipiprant indicated it was not sufficiently efficacious to continue its clinical development. Consequently, the SPIRIT study was terminated early. However, the SPIRIT study offers insight into the long-term safety profile of this DP_2_ receptor antagonist which may be valuable for other molecules in development.

The SPIRIT study found that fevipiprant was well tolerated, with a long-term safety profile similar to placebo. The safety profile was comparable across both doses of fevipiprant in patients treated up to 104 weeks. The key strength of this study is the length of the exposure in a large number of patients (1184 patients had received at least 52 weeks of treatment and 163 patients had received at least 104 weeks of treatment) with a favourable safety profile. This large, long-term safety study provides further evidence for the understanding of the role of DP_2_ receptor antagonists as a potential treatment for asthma.

The exploratory efficacy analysis found an overall reduction in rates of moderate-to-severe exacerbations, an increase in time-to-first moderate-to severe exacerbation and a modest improvement in lung function for fevipiprant versus placebo both in the overall population and the high eosinophil subpopulation. However, the study was terminated early and no multiplicity adjustment was performed for the exploratory analyses. Furthermore, no significant effect on exacerbations was seen in the powered LUSTER studies. Consequently, the exploratory efficacy results from the SPIRIT study should be interpreted with caution.

## Supplementary Information

**Additional file 1: A.** Supplementary figures. **B.** Additional safety results. **C.** Laboratory results. **D.** Additional exploratory analysis for efficacy results. **E.** Inclusion criteria. **F.** Exclusion criteria. **G.** Treatment Period 1 analysis. **H.** Additional statistical methods. **I.** Participating investigators.

## Data Availability

Novartis is committed to sharing with qualified external researchers, access to patient-level data and supporting clinical documents from eligible studies. These requests are reviewed and approved by an independent review panel on the basis of scientific merit. All data provided are anonymised to respect the privacy of patients who have participated in the trial in line with applicable laws and regulations. This trial data availability is according to the criteria and process described on http://www.clinicalstudydatarequest.com.

## Abbreviations

ACQ: Asthma Control Questionnaire
AE: Adverse event
AESI: Adverse events of special interest
AQLQ + 12: Asthma Quality of Life
DP_2_ receptor: Prostaglandin D_2_ receptor 2
EAIR: Exposure-adjusted incidence rate
FAS: Full analysis set
FEV_1_: Forced expiratory volume in one second
GINA: Global Initiative for Asthma
ILC-2: Group 2 innate lymphoid cells
LS: Least squares
mFAS: Modified full analysis set
PG: Prostaglandin
SAE: Serious adverse event
SAF: Safety set
SOC: Standard of care
