# Supplementary material for: Long-term safety and exploratory efficacy of fevipiprant in patients with inadequately controlled asthma: the SPIRIT randomised clinical trial
Source: Respir Res. 2021 Dec 11;22:311. doi: 10.1186/s12931-021-01904-8 (PMC8666007; doi:10.1186/s12931-021-01904-8)
Supplement: Supplementary file 1 — Additional file 1: A. Supplementary figures. B. Additional safety results. C. Laboratory results. D. Additional exploratory analysis for efficacy results. E. Inclusion criteria. F. Exclusion criteria. G. Treatment Period 1 analysis. H. Additional statistical methods. I. Participating investigators. [file 12931_2021_1904_MOESM1_ESM.docx]

# Supporting information

Long-term safety and exploratory efficacy of fevipiprant in patients with inadequately controlled asthma: the SPIRIT randomised clinical trial

*Jorge Maspero, Ioana Octavia Agache, Tadashi Kamei, Makoto Yoshida, Bryan Boone, James M. Felser, Fernando Kawakami, Barbara Knorr, David Lawrence, Thomas Lehmann, Wei Wang and Andrew J. Pedinoff*

Table of Contents

[Supporting information 1](#_Toc65854715)

[A. Supplementary figures 2](#_Toc65854716)

[B. Additional safety results 5](#_Toc65854717)

[C. Laboratory results 26](#_Toc65854718)

[D. Additional exploratory analysis for efficacy results 32](#_Toc65854719)

[E. Inclusion criteria 39](#_Toc65854720)

[F. Exclusion criteria 41](#_Toc65854721)

[G. Treatment Period 1 analysis 52](#_Toc65854722)

[H. Additional statistical methods 62](#_Toc65854723)

[I. Participating investigators 63](#_Toc65854724)

## A Supplementary figures

### Figure S1. Patient disposition


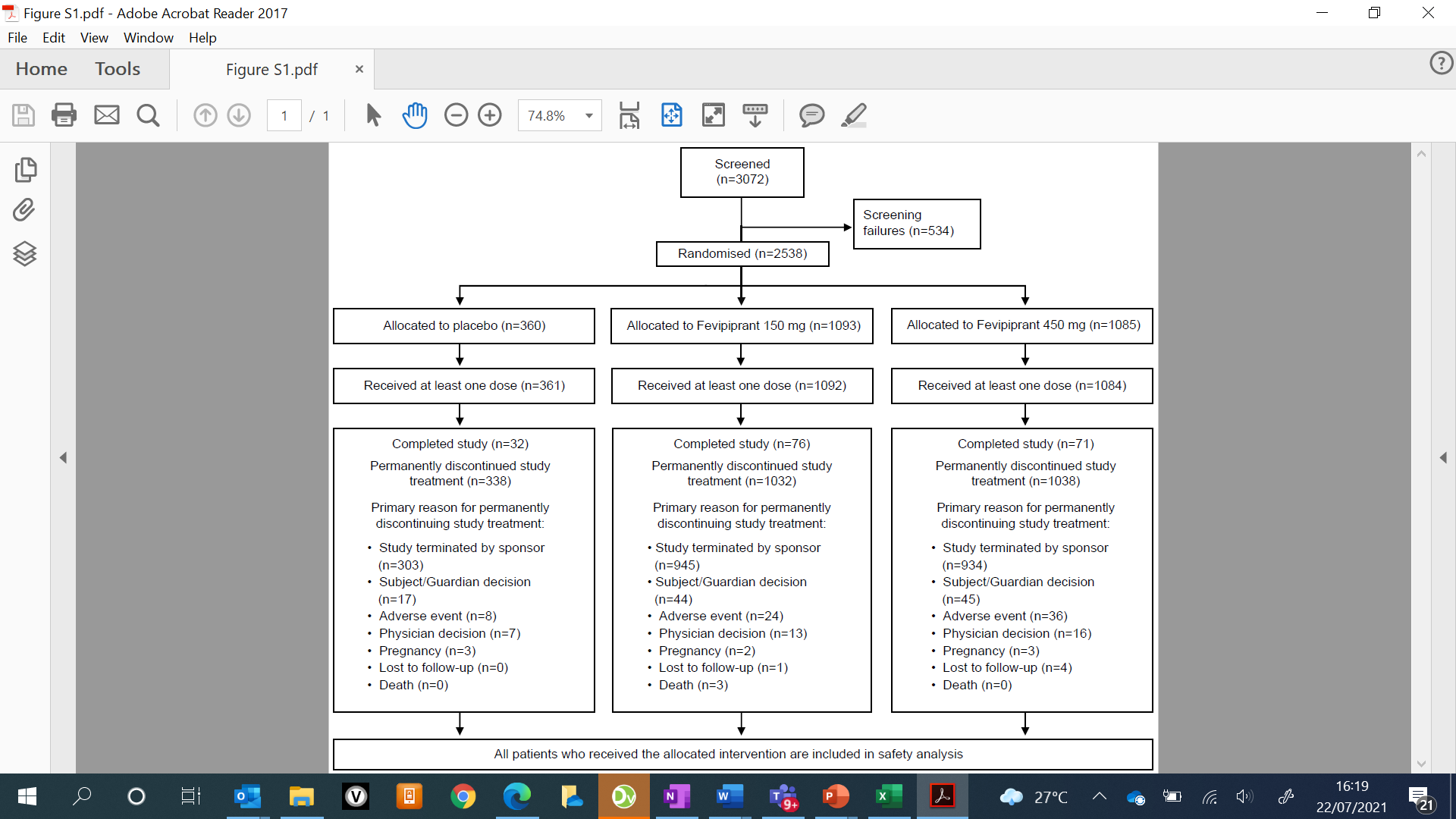


### Figure S2. On-treatment analysis of rate of moderate-to-severe asthma exacerbations during the total treatment period


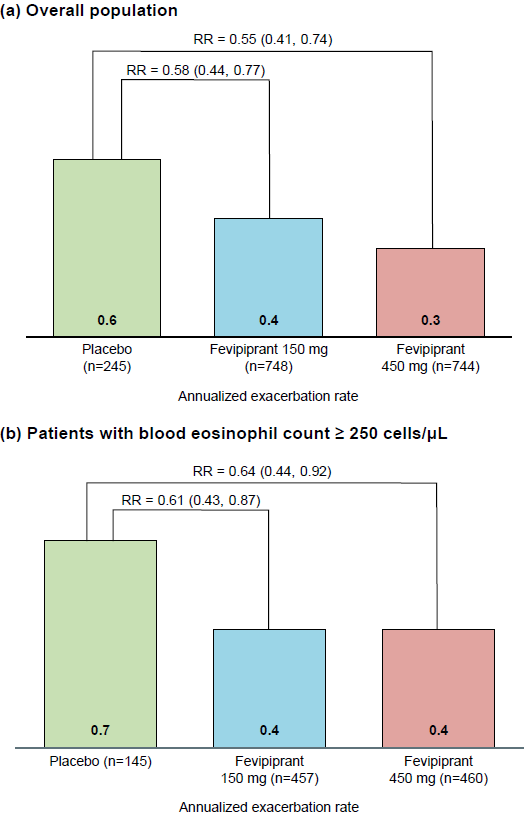


### Figure S3. Kaplan-Meier plot of time to first on-treatment moderate-to-severe asthma exacerbation by population


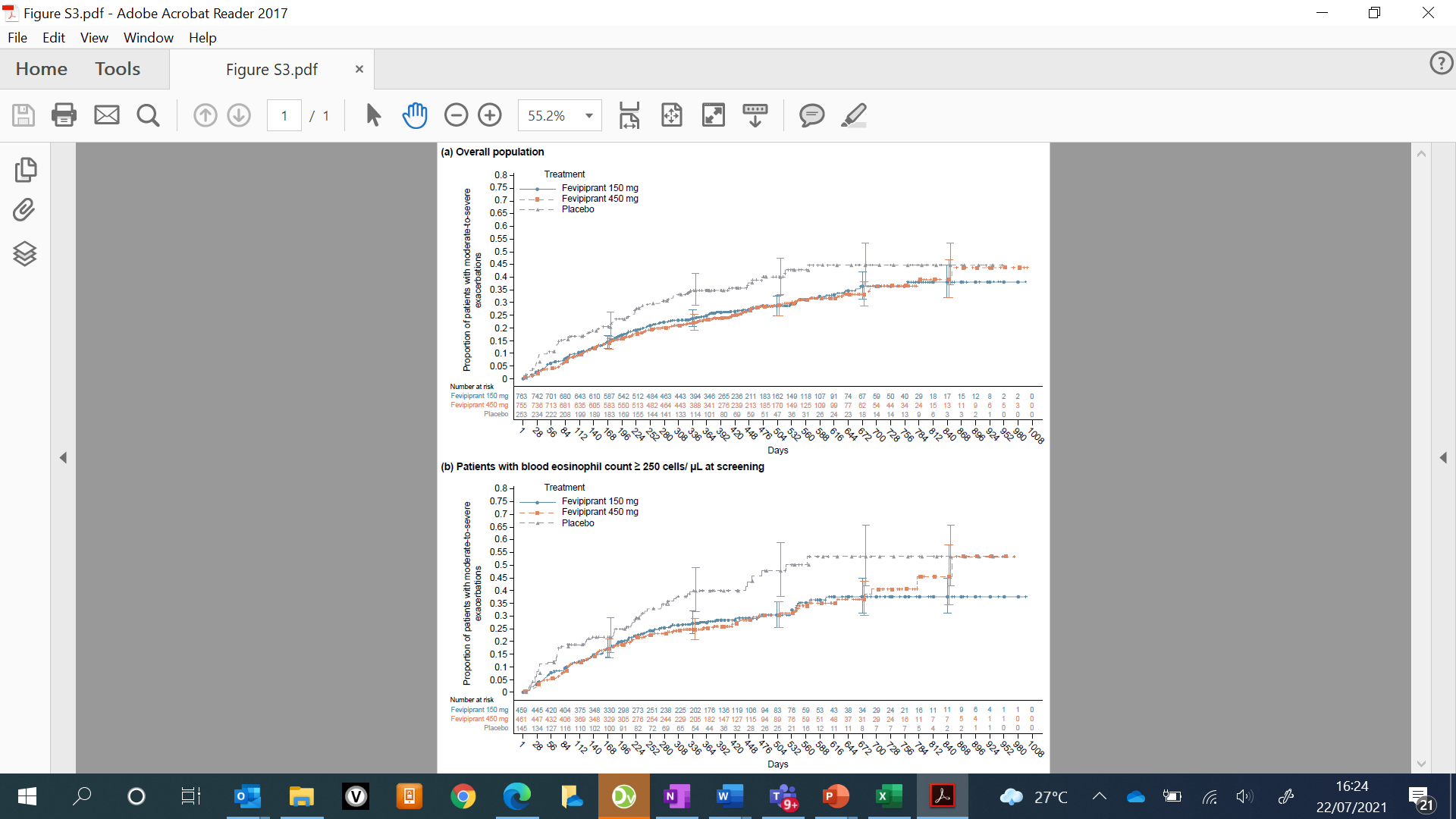


## Additional safety results

### Table S1. Exposure to study treatment

| **Duration of exposure** | **Fevipiprant**  **150 mg**  **N=1092** | **Fevipiprant**  **450 mg**  **N=1084** | **Placebo**  **N=361** | **Total**  **N=2537** |
| --- | --- | --- | --- | --- |
| **Exposure in weeks** | | | | |
| **Mean** | 52.6 | 52.0 | 52.6 | 52.3 |
| **SD** | 28.77 | 28.83 | 29.04 | 28.82 |
| **Minimum** | 0 | 0 | 0 | 0 |
| **Maximum** | 142 | 142 | 141 | 142 |
| **Patient-time (patient-year)** | 1100.8 | 1080.2 | 364.1 | 2545.1 |
| **Cumulative exposure categories – n (%)** | | | | |
| **≥ 1 day** | 1092 (100) | 1084 (100) | 361 (100) | 2537 (100) |
| **≥ 4 weeks** | 1085 (99.4) | 1073 (99.0) | 355 (98.3) | 2513 (99.1) |
| **≥ 12 weeks** | 1058 (96.9) | 1044 (96.3) | 349 (96.7) | 2451 (96.6) |
| **≥ 26 weeks** | 867 (79.4) | 860 (79.3) | 282 (78.1) | 2009 (79.2) |
| **≥ 52 weeks** | 515 (47.2) | 497 (45.8) | 172 (47.6) | 1184 (46.7) |
| **≥ 104 weeks** | 74 (6.8) | 65 (6.0) | 24 (6.6) | 163 (6.4) |

Duration of exposure = Date of last dose of study drug in the treatment period - date of first dose of study drug +1. SD = standard deviation.

### Table S2. Exposure adjusted incidence rates of treatment emergent adverse events by primary system organ class – subgroup age

|  | **< 18 years** | | | **18 < 65 years** | | | **≥ 65 years** | | |
| --- | --- | --- | --- | --- | --- | --- | --- | --- | --- |
| **Primary system organ class** | **Fevipiprant 150 mg**  **N=48**  **exp. = 31.0 PY**  **n**  **IR** | **Fevipiprant 450 mg**  **N=53**  **exp.= 33.4 PY**  **n**  **IR** | **Placebo**  **N= 17**  **exp.= 10.5 PY**  **n**  **IR** | **Fevipiprant 150 mg**  **N= 855**  **exp.= 877.5 PY**  **n**  **IR** | **Fevipiprant**  **450 mg**  **N=835**  **exp.= 848.1 PY**  **n**  **IR** | **Placebo**  **N= 281**  **exp.= 292.4 PY**  **n**  **IR** | **Fevipiprant**  **150 mg**  **N=189**  **exp.=192.3 PY**  **n**  **IR** | **Fevipiprant 450 mg**  **N= 196**  **exp. =198.7 PY**  **n**  **IR** | **Placebo**  **N= 63**  **exp.= 61.2 PY**  **n**  **IR** |
| Number of patients with at least one AE | 29  93.6 | 26  77.9 | 8  76.3 | 550  62.7 | 529  62.4 | 195  66.7 | 137  71.2 | 131  65.9 | 42  68.6 |
| Blood and lymphatic system disorders | 0  0 | 1  3.0 | 0  0 | 17  1.9 | 7  0.8 | 5  1.7 | 6  3.1 | 4  2.0 | 0  0 |
| Cardiac disorders | 0  0 | 0  0 | 0  0 | 8  0.9 | 21  2.5 | 6  2.1 | 8  4.2 | 13  6.5 | 1  1.6 |
| Congenital, familial and genetic disorders | 0  0 | 1  3.0 | 0  0 | 1  0.1 | 1  0.1 | 0  0 | 0  0 | 1  0.5 | 1  1.6 |
| Ear and labyrinth disorders | 0  0 | 0  0 | 1  9.5 | 16  1.8 | 12  1.4 | 7  2.4 | 2  1.0 | 1  0.5 | 1  1.6 |
| Endocrine disorders | 1  3.2 | 0  0 | 0  0 | 3  0.3 | 5  0.6 | 0 | 1  0.5 | 2  1.0 | 0  0 |
| Eye disorders | 1  3.2 | 0  0 | 0  0 | 15  1.7 | 10  1.2 | 7  2.4 | 2  1.0 | 4  2.0 | 2  3.3 |
| Gastrointestinal disorders | 6  19.4 | 2  6.0 | 2  19.1 | 75  8.5 | 71  8.4 | 26  8.9 | 20  10.4 | 24  12.1 | 10  16.3 |
| General disorders and administration site conditions | 0  0 | 1  3.0 | 0  0 | 34  3.9 | 25  2.9 | 6  2.1 | 9  4.7 | 8  4.0 | 2  3.3 |
| Hepatobiliary disorders | 0  0 | 0  0 | 1  9.5 | 7  0.8 | 16  1.9 | 3  1.0 | 3  1.6 | 5  2.5 | 4  6.5 |
| Immune system disorders | 2  6.5 | 0  0 | 1  9.5 | 8  0.9 | 9  1.1 | 5  1.7 | 1  0.5 | 2  1.0 | 0  0 |
| Infections and infestations | 13  42.0 | 15  45.0 | 6  57.2 | 350  39.9 | 323  38.1 | 123  42.1 | 76  39.5 | 80  40.3 | 22  35.9 |
| Injury, poisoning and procedural complications | 1  3.2 | 2  6.0 | 1  9.5 | 62  7.1 | 42  5.0 | 23  7.9 | 19  9.9 | 19  9.6 | 5  8.2 |
| Investigations | 2  6.5 | 2  6.0 | 1  9.5 | 71  8.1 | 79  9.3 | 19  6.5 | 19  9.9 | 21  10.6 | 4  6.5 |
| Metabolism and nutrition disorders | 0  0 | 3  9.0 | 0  0 | 51  5.8 | 53  6.2 | 19  6.5 | 12  6.2 | 5  2.5 | 5  8.2 |
| Musculoskeletal and connective tissue disorders | 1  3.2 | 1  3.0 | 1  9.5 | 80  9.1 | 81  9.6 | 24  8.2 | 21  10.9 | 11  5.5 | 6  9.8 |
| Neoplasms benign, malignant and unspecified (incl cysts and polyps) | 0  0 | 0  0 | 0  0 | 10  1.1 | 7  0.8 | 1  0.3 | 7  3.6 | 4  2.0 | 2  3.3 |
| Nervous system disorders | 4  12.9 | 3  9.0 | 2  19.1 | 64  7.3 | 60  7.1 | 24  8.2 | 16  8.3 | 15  7.6 | 6  9.8 |
| Product issues | 0  0 | 0  0 | 0  0 | 0  0 | 0  0 | 0  0 | 1  0.5 | 0  0 | 0  0 |
| Pregnancy, puerperium and perinatal conditions | 0  0 | 0  0 | 0  0 | 1  0.1 | 0  0 | 0  0 | 0  0 | 0  0 | 0  0 |
| Psychiatric disorders | 1  3.2 | 0  0 | 0  0 | 17  1.9 | 14  1.7 | 7  2.4 | 6  3.1 | 4  2.0 | 2  3.3 |
| Renal and urinary disorders | 1  3.2 | 0  0 | 1  9.5 | 27  3.1 | 35  4.1 | 8  2.7 | 9  4.7 | 10  5.0 | 1  1.6 |
| Reproductive system and breast disorders | 0  0 | 0  0 | 0  0 | 15  1.7 | 9  1.1 | 4  1.4 | 3  1.6 | 1  0.5 | 0  0 |
| Respiratory, thoracic and mediastinal disorders | 16  51.7 | 14  42.0 | 5  47.7 | 265  30.2 | 264  31.1 | 117  40.0 | 57  29.6 | 65  32.7 | 22  35.9 |
| Skin and subcutaneous tissue disorders | 2  6.5 | 0 | 0 | 33  3.8 | 38  4.5 | 14  4.8 | 10  5.2 | 9  4.5 | 1  1.6 |
| Social circumstances | 0  0 | 0  0 | 0  0 | 1  0.1 | 1  0.1 | 3  1.0 | 0  0 | 0  0 | 0  0 |
| Vascular disorders | 0  0 | 0  0 | 0  0 | 30  3.4 | 30  3.5 | 12  4.1 | 15  7.8 | 10  5.0 | 1  1.6 |

A patient with multiple AEs is counted only once in the “Number of patients with at least one AE” row. A patient with multiple adverse events within a primary system organ class is counted only once for that primary system organ class. AE = adverse event; exp. = overall exposure time in person-years (PY); n = number of patients with events; IR = incidence rate per 100 person-years. System organ classes are presented in alphabetical order. MedDRA Version 22.1 has been used for the reporting of adverse events.

### Table S3. Exposure adjusted incidence rates of treatment emergent adverse events by primary system organ class – subgroup sex

|  | **Male** | | | **Female** | | |
| --- | --- | --- | --- | --- | --- | --- |
| **Primary system organ class** | **Fevipiprant 150 mg**  **N=433**  **exp.=447.7 PY**  **n**  **IR** | **Fevipiprant 450 mg**  **N=418**  **exp.=425.6 PY**  **n**  **IR** | **Placebo**  **N=132**  **exp.=143.1 PY**  **n**  **IR** | **Fevipiprant**  **150 mg**  **N=659**  **exp.= 653.1 PY**  **n**  **IR** | **Fevipiprant**  **450 mg**  **N=666**  **exp.=654.6 PY**  **n**  **IR** | **Placebo**  **N=229**  **exp.= 221.0 PY**  **n**  **IR** |
| Number of patients with at least one AE | 275  61.4 | 264  62.0 | 87  60.8 | 441  67.5 | 422  64.5 | 158  71.5 |
| Blood and lymphatic system disorders | 7  1.6 | 3  0.7 | 2  1.4 | 16  2.4 | 9  1.4 | 3  1.4 |
| Cardiac disorders | 8  1.8 | 21  4.9 | 2  1.4 | 8  1.2 | 13  2.0 | 5  2.3 |
| Congenital, familial and genetic disorders | 1  0.2 | 1  0.2 | 1  0.7 | 0  0 | 2  0.3 | 0  0 |
| Ear and labyrinth disorders | 3  0.7 | 3  0.7 | 3  2.1 | 15  2.3 | 10  1.5 | 6  2.7 |
| Endocrine disorders | 4  0.9 | 2  0.5 | 0  0 | 1  0.2 | 5  0.8 | 0  0 |
| Eye disorders | 8  1.8 | 7  1.6 | 8  5.6 | 10  1.5 | 7  1.1 | 1  0.5 |
| Gastrointestinal disorders | 37  8.3 | 39  9.2 | 14  9.8 | 64  9.8 | 58  8.9 | 24  10.9 |
| General disorders and administration site conditions | 21  4.7 | 12  2.8 | 3  2.1 | 22  3.4 | 22  3.4 | 5  2.3 |
| Hepatobiliary disorders | 2  0.4 | 10  2.3 | 4  2.8 | 8  1.2 | 11  1.7 | 4  1.8 |
| Immune system disorders | 6  1.3 | 1  0.2 | 2  1.4 | 5  0.8 | 10  1.5 | 4  1.8 |
| Infections and infestations | 168  37.5 | 150  35.2 | 46  32.1 | 271  41.5 | 268  40.9 | 105  47.5 |
| Injury, poisoning and procedural complications | 28  6.3 | 22  5.2 | 10  7.0 | 54  8.3 | 41  6.3 | 19  8.6 |
| Investigations | 38  8.5 | 56  13.2 | 12  8.4 | 54  8.3 | 46  7.0 | 12  5.4 |
| Metabolism and nutrition disorders | 34  7.6 | 25  5.9 | 8  5.6 | 29  4.4 | 36  5.5 | 12  7.2 |
| Musculoskeletal and connective tissue disorders | 45  10.1 | 32  7.5 | 11  7.7 | 57  8.7 | 61  9.3 | 20  9.0 |
| Neoplasms, benign, malignant and unspecified (incl cysts and polyps) | 7  1.6 | 5  1.2 | 2  1.4 | 10  1.5 | 6  0.9 | 1  0.5 |
| Nervous system disorders | 25  5.6 | 24  5.6 | 10  7.0 | 59  9.0 | 54  8.2 | 22  10.0 |
| Pregnancy, puerperium and perinatal conditions | 0  0 | 0  0 | 0  0 | 1  0.2 | 0  0 | 0  0 |
| Product issues | 0  0 | 0  0 | 0  0 | 1  0.2 | 0  0 | 0  0 |
| Psychiatric disorders | 7  1.6 | 5  1.2 | 5  3.5 | 17  2.6 | 13  2.0 | 4  1.8 |
| Renal and urinary disorders | 11  2.5 | 22  5.2 | 5  3.5 | 26  4.0 | 23  3.5 | 5  2.3 |
| Reproductive system and breast disorders | 5  1.1 | 7  1.6 | 0  0 | 13  2.0 | 3  0.5 | 4  1.8 |
| Respiratory, thoracic and mediastinal disorders | 118  26.4 | 109  25.6 | 45  31.4 | 220  33.7 | 234  35.7 | 99  44.8 |
| Skin and subcutaneous tissue disorders | 13  2.9 | 23  5.4 | 6  4.2 | 32  4.9 | 24  3.7 | 9  4.1 |
| Social circumstances | 0  0 | 0  0 | 0  0 | 1  0.2 | 1  0.2 | 3  1.4 |
| Vascular disorders | 14  3.1 | 17  4.0 | 6  4.2 | 31  4.7 | 23  3.5 | 7  3.2 |

A patient with multiple AEs is counted only once in the “Number of patients with at least one AE” row. A patient with multiple adverse events within a primary system organ class is counted only once for that primary system organ class. AE = adverse event; exp. = overall exposure time in person-years (PY); n = number of patients with events; IR = incidence rate per 100 person-years. System organ classes are presented in alphabetical order. MedDRA Version 22.1 has been used for the reporting of adverse events.

### Table S4. Exposure adjusted incidence rates of treatment emergent adverse events by primary system organ class – subgroup age of onset of asthma

|  | **< 18 years** | | | **≥ 18 years** | | |
| --- | --- | --- | --- | --- | --- | --- |
| **Primary system organ class** | **Fevipiprant 150 mg**  **N=340**  **exp.=333.7 PY**  **n**  **IR** | **Fevipiprant 450 mg**  **N=339**  **exp.=327.7 PY**  **n**  **IR** | **Placebo**  **N=99**  **exp. = 96.9 PY**  **n**  **IR** | **Fevipiprant 150 mg**  **N=750**  **exp.=764.7 PY**  **n**  **IR** | **Fevipiprant**  **450 mg**  **N=745**  **exp.= 752.5 PY**  **n**  **IR** | **Placebo**  **N=262**  **exp=267.2 PY**  **n**  **IR** |
| Number of patients with at least one AE | 221  66.2 | 223  68.1 | 67  69.1 | 493  64.5 | 463  61.5 | 178  66.6 |
| Blood and lymphatic system disorders | 5  1.5 | 2  0.6 | 1  1.0 | 17  2.2 | 10  1.3 | 4  1.5 |
| Cardiac disorders | 0  0 | 5  1.5 | 2  2.1 | 16  2.1 | 29  3.9 | 5  1.9 |
| Congenital, familial and genetic disorders | 0  0 | 2  0.6 | 0  0 | 1  0.1 | 1  0.1 | 1  0.4 |
| Ear and labyrinth disorders | 5  1.5 | 5  1.5 | 2  2.1 | 13  1.7 | 8  1.1 | 7  2.6 |
| Endocrine disorders | 1  0.3 | 2  0.6 | 0  0 | 4  0.5 | 5  0.7 | 0  0 |
| Eye disorders | 3  0.9 | 4  1.2 | 3  3.1 | 15  2.0 | 10  1.3 | 6  2.2 |
| Gastrointestinal disorders | 25  7.5 | 36  11.0 | 10  10.3 | 76  9.9 | 61  8.1 | 28  10.5 |
| General disorders and administration site conditions | 13  3.9 | 9  2.7 | 1  1.0 | 30  3.9 | 25  3.3 | 7  2.6 |
| Hepatobiliary disorders | 3  0.9 | 6  1.8 | 5  5.2 | 7  0.9 | 15  2.0 | 3  1.1 |
| Immune system disorders | 2  0.6 | 4  1.2 | 2  2.1 | 9  1.2 | 7  0.9 | 4  1.5 |
| Infections and infestations | 137  41.1 | 131  40.0 | 43  44.4 | 300  39.2 | 287  38.1 | 108  40.4 |
| Injury, poisoning and procedural complications | 23  6.9 | 19  5.8 | 6  6.2 | 58  7.6 | 44  5.8 | 23  8.6 |
| Investigations | 24  7.2 | 22  6.7 | 6  6.2 | 68  8.9 | 80  10.6 | 18  6.7 |
| Metabolism and nutrition disorders | 17  5.1 | 20  6.1 | 5  5.2 | 46  6.0 | 41  5.4 | 19  7.1 |
| Musculoskeletal and connective tissue disorders | 31  9.3 | 37  11.3 | 9  9.3 | 71  9.3 | 56  7.4 | 22  8.2 |
| Neoplasms benign, malignant and unspecified (incl cysts and polyps) | 1  0.3 | 6  1.8 | 0  0 | 16  2.1 | 5  0.7 | 3  1.1 |
| Nervous system disorders | 24  7.2 | 25  7.6 | 8  8.3 | 58  7.6 | 53  7.0 | 24  9.0 |
| Pregnancy, puerperium and perinatal conditions | 1  0.3 | 0  0 | 0  0 | 1  0.1 | 0  0 | 0  0 |
| Product issues | 0  0 | 0  0 | 0  0 | 1  0.1 | 0  0 | 0  0 |
| Psychiatric disorders | 10  3.0 | 3  0.9 | 4  4.1 | 14  1.8 | 15  2.0 | 5  1.9 |
| Renal and urinary disorders | 16  4.8 | 15  4.6 | 5  5.2 | 21  2.7 | 30  4.0 | 5  1.9 |
| Reproductive system and breast disorders | 7  2.1 | 1  0.3 | 2  2.1 | 11  1.4 | 9  1.2 | 2  0.7 |
| Respiratory, thoracic and mediastinal disorders | 104  31.2 | 110  33.6 | 34  35.1 | 233  30.5 | 233  31.0 | 110  41.2 |
| Skin and subcutaneous tissue disorders | 13  3.9 | 18  5.5 | 6  6.2 | 32  4.2 | 29  3.9 | 9  3.4 |
| Social circumstances | 1  0.3 | 1  0.3 | 0  0 | 0  0 | 0  0 | 3  1.1 |
| Vascular disorders | 5  1.5 | 14  4.3 | 3  3.1 | 38  5.0 | 26  3.5 | 10  3.7 |

A patient with multiple AEs is counted only once in the “Number of patients with at least one AE” row. A patient with multiple adverse events within a primary system organ class is counted only once for that primary system organ class. AE = adverse event; exp. = overall exposure time in person-years (PY); n = number of patients with events; IR = incidence rate per 100 person-years. System organ classes are presented in alphabetical order. MedDRA Version 22.1 has been used for the reporting of adverse events.

### Table S5. Exposure adjusted incidence rate for most frequent treatment emergent adverse events (at least 2.0 per 100 person-years in any treatment group) by Preferred Term

| **Preferred term** | **Fevipiprant 150 mg**  **(N=1092)**  **Exp.=1100.8 PY**  **n**  **IR** | **Fevipiprant 450 mg**  **(N=1084)**  **Exp.=1080.2 PY**  **n**  **IR** | **Placebo**  **(N=361)**  **Exp.=364.1 PY**  **n**  **IR** |
| --- | --- | --- | --- |
| **Number of patients with at least one AE** | 716  65.0 | 686  63.5 | 245  67.3 |
| **Asthma** | 298  27.1 | 283  26.2 | 129  35.4 |
| **Nasopharyngitis** | 110  10.0 | 106  9.8 | 36  9.9 |
| **Bronchitis** | 75  6.8 | 58  5.4 | 38  10.4 |
| **Upper respiratory tract infection** | 72  6.5 | 43  4.0 | 25  6.9 |
| **Headache** | 42  3.8 | 29  2.7 | 24  6.6 |
| **Sinusitis** | 39  3.5 | 28  2.6 | 9  2.5 |
| **Pharyngitis** | 34  3.1 | 22  2.0 | 15  4.1 |
| **Viral upper respiratory tract infection** | 34  3.1 | 32  3.0 | 9  2.5 |
| **Back pain** | 31  2.8 | 31  2.9 | 10  2.7 |
| **Hypertension** | 31  2.8 | 31  2.9 | 11  3.0 |
| **Influenza** | 31  2.8 | 29  2.7 | 9  2.5 |
| **Blood creatinine increased** | 30  2.7 | 42  3.9 | 2  0.5 |
| **Arthralgia** | 23  2.1 | 11  1.0 | 8  2.2 |
| **Urinary tract infection** | 23  2.1 | 37  3.4 | 12  3.3 |
| **Rhinitis allergic** | 19  1.7 | 28  2.6 | 11  3.0 |
| **Upper respiratory tract infection bacterial** | 14  1.3 | 32  3.0 | 12  3.3 |

AE = adverse event; exp. = overall exposure time in person-years (PY); IR = incidence rate per 100 person-years; n = number of patients with events.

A patient with multiple AEs is counted only once in the “Number of patients with at least one AE” row. A patient with multiple AEs within a preferred term is counted only once for that preferred term. Preferred terms are in descending order of AE IR in the fevipiprant 150 mg group.

MedDRA Version 22.1 has been used for the reporting of AEs.

### Table S6. Exposure adjusted incidence rates of treatment emergent AEs by primary system organ class SOC

| **Primary system organ class** | **Fevipiprant 150 mg (N=1092)**  **Exp.=1100.8 PY**  **n**  **IR** | **Fevipiprant 450 mg (N=1084)**  **Exp.=1080.2 PY**  **n**  **IR** | **Placebo**  **(N=361)**  **Exp.=364.1 PY**  **n**  **IR** |
| --- | --- | --- | --- |
| **Number of patients with at least one AE** | 716  65.0 | 686  63.5 | 245  67.3 |
| **Blood and lymphatic system disorders** | 23  2.1 | 12  1.1 | 5  1.4 |
| **Cardiac disorders** | 16  1.5 | 34  3.1 | 7  1.9 |
| **Congenital, familial and genetic disorders** | 1  0.1 | 3  0.3 | 1  0.3 |
| **Ear and labyrinth disorders** | 18  1.6 | 13  1.2 | 9  2.5 |
| **Endocrine disorders** | 5  0.5 | 7  0.6 | 0  0 |
| **Eye disorders** | 18  1.6 | 14  1.3 | 9  2.5 |
| **Gastrointestinal disorders** | 101  9.2 | 97  9.0 | 38  10.4 |
| **General disorders and administrative site conditions** | 43  3.9 | 34  3.1 | 8  2.2 |
| **Hepatobiliary disorders** | 10  0.9 | 21  1.9 | 8  2.2 |
| **Immune system disorders** | 11  1.0 | 11  1.0 | 6  1.6 |
| **Infections and infestations** | 439  39.9 | 418  38.7 | 151  41.5 |
| **Injury, poisoning and procedural complications** | 82  7.4 | 63  5.8 | 29  8.0 |
| **Investigations** | 92  8.4 | 102  9.4 | 24  6.6 |
| **Metabolism and nutrition disorders** | 63  5.7 | 61  5.6 | 24  6.6 |
| **Musculoskeletal and connective tissue disorders** | 102  9.3 | 93  8.6 | 31  8.5 |
| **Neoplasms benign, malignant and unspecified (including cysts and polyps)** | 17  1.5 | 11  1.0 | 3  0.8 |
| **Nervous system disorders** | 84  7.6 | 78  7.2 | 32  8.8 |
| **Pregnancy, puerperium and perinatal conditions** | 1  0.1 | 0  0 | 0  0 |
| **Product issues** | 1  0.1 | 0  0 | 0  0 |
| **Psychiatric disorders** | 24  2.2 | 18  1.7 | 9  2.5 |
| **Renal and urinary disorders** | 37  3.4 | 45  4.2 | 10  2.7 |
| **Reproductive system and breast disorders** | 18  1.6 | 10  0.9 | 4  1.1 |
| **Respiratory, thoracic and mediastinal disorders** | 338  30.7 | 343  31.8 | 144  39.5 |
| **Skin and subcutaneous tissue disorders** | 45  4.1 | 47  4.4 | 15  4.1 |
| **Social circumstances** | 1  0.1 | 1  0.1 | 3  0.8 |
| **Vascular disorders** | 45  4.1 | 40  3.7 | 13  3.6 |

AE = adverse event; exp.=overall exposure time in person-years (PY); IR=incidence rate per 100 person-years; n=number of patients with events.

A patient with multiple AEs is counted only once in the “Number of patients with at least one AE” row. A patient with multiple AEs within a primary system organ class is counted only once for that primary system organ class. System organ classes are presented in alphabetical order.

MedDRA Version 22.1 has been used for the reporting of adverse events.

### Table S7. Exposure adjusted incidence rate for most frequent treatment emergent serious adverse events (at least 0.2 per 100 person-years in any treatment group) by Preferred Term

| **Preferred term** | **Fevipiprant 150 mg (N=1092)**  **Exp.=1100.8 PY**  **n**  **IR** | **Fevipiprant 450 mg (N=1084)**  **Exp.=1080.2 PY**  **n**  **IR** | **Placebo (N=361)**  **Exp.=364.1 PY**  **n**  **IR** |
| --- | --- | --- | --- |
| **Number of patients with at least one SAE** | 87  7.9 | 64  5.9 | 33  9.1 |
| **Asthma** | 30  2.7 | 16  1.5 | 13  3.6 |

exp.=overall exposure time in person-years (PY); IR = incidence rate per 100 person-years; n=number of patients with events; SAE, serious adverse event.

A patient with multiple SAEs is counted only once in the “Number of patients with at least one SAE” row. A patient with multiple SAEs within a preferred term is counted only once for that preferred term. Preferred terms are in descending order of SAE IR in the fevipiprant 150 mg group. MedDRA Version 22.1 has been used for the reporting of AEs.

### Table S8. Exposure adjusted incidence rate of treatment emergent adverse events leading to study treatment discontinuation by primary system organ class

| **Primary system organ class** | **Fevipiprant 150 mg**  **(N=1092)**  **Exp.=1100.8 PY**  **n**  **IR** | **Fevipiprant 450 mg**  **(N=1084)**  **Exp.=1080.2 PY**  **n**  **IR** | **Placebo**  **(N=361)**  **Exp.=364.1 PY**  **n**  **IR** |
| --- | --- | --- | --- |
| **Number of patients with at least one AE** | 30  2.7 | 37  3.4 | 9  2.5 |
| **Blood and lymphatic system disorders** | 0  0 | 1  0.1 | 0  0 |
| **Cardiac disorders** | 2  0.2 | 7  0.6 | 0  0 |
| **Ear and labyrinth disorders** | 0  0 | 1  0.1 | 0  0 |
| **Eye disorders** | 1  0.1 | 0  0 | 0  0 |
| **Gastrointestinal disorders** | 5  0.5 | 6  0.6 | 2  0.5 |
| **General disorders and administrative site conditions** | 1  0.1 | 0  0 | 0  0 |
| **Hepatobiliary disorders** | 0  0 | 2  0.2 | 1  0.3 |
| **Immune system disorders** | 0  0 | 1  0.1 | 0  0 |
| **Infections and infestations** | 3  0.3 | 1  0.1 | 1  0.3 |
| **Injury, poisoning and procedural complications** | 1  0.1 | 1  0.1 | 0  0 |
| **Investigations** | 6  0.5 | 6  0.6 | 1  0.3 |
| **Metabolism and nutrition disorders** | 0  0 | 0  0 | 1  0.3 |
| **Musculoskeletal and connective tissue disorders** | 1  0.1 | 1  0.1 | 1  0.3 |
| **Neoplasms benign, malignant and unspecified (including cysts and polyps)** | 4  0.4 | 3  0.3 | 1  0.3 |
| **Nervous system disorders** | 2  0.2 | 4  0.4 | 2  0.5 |
| **Pregnancy, puerperium and perinatal conditions** | 1  0.1 | 0  0 | 0  0 |
| **Psychiatric disorders** | 2  0.2 | 0  0 | 0  0 |
| **Renal and urinary disorders** | 2  0.2 | 1  0.1 | 0  0 |
| **Respiratory, thoracic and mediastinal disorders** | 3  0.3 | 2  0.2 | 1  0.3 |
| **Skin and subcutaneous tissue disorders** | 2  0.2 | 3  0.3 | 0  0 |
| **Social circumstances** | 0  0 | 0  0 | 1  0.3 |
| **Vascular disorders** | 0  0 | 2  0.2 | 0  0 |

AE = adverse event; exp. = overall exposure time in person-years (PY); IR = incidence rate per 100 person-years; n = number of patients with events.

A patient with multiple AEs is counted only once in the “Number of patients with at least one AE” row. A patient with multiple AEs within a primary system organ class is counted only once for that primary system organ class. System organ classes are presented in alphabetical order.

MedDRA Version 22.1 has been used for the reporting of adverse events.

### Adverse events of special interest (AESI)

Cardiac AESI were reported with a higher EAIR for fevipiprant 450 mg group (IR=3.1) compared with fevipiprant 150 mg (IR=1.7) and placebo (IR=2.2) groups. Tachycardia AESI were reported with a lower EAIR for fevipiprant 150 mg group (IR=0.8) compared with fevipiprant 450 mg (IR=1.4) and placebo (IR=1.4) groups. Hepatotoxicity AESI were reported with a higher EAIR in placebo group (IR=3.0) compared with fevipiprant 150 mg (IR=1.3) and fevipiprant 450 mg (IR=1.7). Most events were laboratory abnormalities. Idiosyncratic drug reactions AESI were reported with comparable EAIR across the treatment groups (1.2 in fevipiprant 150 mg, 0.9 in fevipiprant 450 mg, and 0.8 in placebo).

### Treatment emergent deaths

There were five treatment-emergent deaths reported in the study. Three patients died in the fevipiprant 150 mg group, with associated serious adverse events of cardiac failure; cellulitis, septic shock; and dengue haemorrhagic fever, pneumonia, septic shock. One patient died in the fevipiprant 450 mg group with associated serious adverse event of colloid brain cyst and there was one sudden death in the placebo treatment group.

## C. Laboratory results

### **Table S9. Number (%) of patients with newly occurring or worsening clinically notable on-treatment hematology values at any time post-baseline using SPIRIT baseline**

| **Variable** | **Notable criteria** | **Fevipiprant**  **150 mg**  **N=1092**  **n/m (%)** | **Fevipiprant**  **450 mg**  **N=1084**  **n/m (%)** | **Placebo**  **N=361**  **n/m (%)** |
| --- | --- | --- | --- | --- |
| **Hematocrit (v/v)** | Total | 24/1067 (2.2) | 15/1068 (1.4) | 6/355 (1.7) |
|  | < 0.34 (Male aged 12-17) | 0/ 27 (0) | 0/ 25 (0) | 0/ 12 (0) |
|  | < 0.37 (Male aged 18-65) | 8/ 340 (2.4) | 6/ 325 (1.8) | 3/98 (3.1) |
|  | < 0.34 (Male aged ≥ 66) | 1/ 53 (1.9) | 1/ 62 (1.6) | 0/ 21 (0) |
|  | < 0.32 (Female aged 12-65) | 14/ 543 (2.6) | 4/ 552 (0.7) | 3/ 196 (1.5) |
|  | < 0.31 (Female aged ≥ 66) | 1/ 104 (1.0) | 4/ 104 (3.8) | 0/ 28 (0) |
| **Haemoglobin (g/L)** | Total | 14/1075 (1.3) | 9/1070 (0.8) | 3/ 356 (0.8) |
|  | < 100 (Male aged 12-17) | 0/ 27 (0) | 0/ 25 (0) | 0/ 12 (0) |
|  | < 110 (Male aged ≥ 18) | 3/ 398 (0.8) | 3/ 387 (0.8) | 2/ 119 (1.7) |
|  | < 95 (Female) | 11/ 650 (1.7) | 6/ 658 (0.9) | 1/ 225 (0.4) |
| **Platelets (10E9/L)** | Total | 1/1072 (0.1) | 2/1066 (0.2) | 1/ 356 (0.3) |
|  | < 75 | 0/1072 (0) | 2/1066 (0.2) | 0/ 356 (0) |
|  | > 700 | 1/1072 (0.1) | 0/1066 (0) | 1/ 356 (0.3) |
| **Leukocytes (10E9/L)** | Total | 22/1075 (2.0) | 9/1067 (0.8) | 2/ 356 (0.6) |
|  | < 2.8 | 7/1075 (0.7) | 2/1067 (0.2) | 0/ 356 (0) |
|  | > 16 | 15/1075 (1.4) | 7/1067 (0.7) | 2/ 356 (0.6) |

m, number of patients with a post-baseline value for the specified category; n, number of patients meeting the criterion, i.e. who had a newly occurring clinically notable value or had a worsening of a value during treatment which was already notable at baseline; N, Total number of patients in the treatment group in this analysis set. SPIRIT baseline is used as the baseline value for all patients. For patients with a missing value at baseline, any post-baseline notable value is considered as newly occurring.

### Table S10. Number (%) of patients with newly occurring or worsening clinically notable on-treatment biochemistry values at any time post-baseline using SPIRIT baseline

| **Variable** | **Notable criteria** | **Fevipiprant**  **150 mg**  **N=1092**  **n/m (%)** | **Fevipiprant**  **450 mg**  **N=1084**  **n/m (%)** | **Placebo**  **N=361**  **n/m (%)** |
| --- | --- | --- | --- | --- |
| Alanine Aminotransferase (U/L) | Total | 9/1075 (0.8) | 6/1071 (0.6) | 6/ 356 (1.7) |
|  | > 3 x ULN | 9/1075 (0.8) | 6/1071 (0.6) | 6/ 356 (1.7) |
| Alkaline Phosphatase (IU/L) | Total | 1/1075 (0.1) | 0/1071 (0) | 2/ 356 (0.6) |
|  | > 3 x ULN | 1/1075 (0.1) | 0/1071 (0) | 2/ 356 (0.6) |
| Aspartate Aminotransferase (U/L) | Total | 5/1075 (0.5) | 5/1071 (0.5) | 4/ 356 (1.1) |
|  | > 3 x ULN | 5/1075 (0.5) | 5/1071 (0.5) | 4/ 356 (1.1) |
| BUN/ Urea (mmol/L) | Total | 22/1076 (2.0) | 23/1071 (2.1) | 3/ 356 (0.8) |
|  | > 9.99 | 22/1076 (2.0) | 23/1071 (2.1) | 3/ 356 (0.8) |
| Creatinine (μmol/L) | Total | 3/1076 (0.3) | 2/1071 (0.2) | 0/ 356 (0) |
|  | > 176.8 | 3/1076 (0.3) | 2/1071 (0.2) | 0/ 356 (0) |
| Gamma Glutamyl Transferase (U/L) | Total | 18/1076 (1.7) | 14/1071 (1.3) | 9/ 356 (2.5) |
|  | > 3 x ULN | 18/1076 (1.7) | 14/1071 (1.3) | 9/ 356 (2.5) |
| Glucose (mmol/L) | Total | 37/1075 (3.4) | 33/1071 (3.1) | 12/356 (3.4) |
|  | < 2.78 | 4/1075 (0.4) | 3/1071 (0.3) | 1/356 (0.3) |
|  | > 9.99 | 33/1075 (3.1) | 30/1071(2.8) | 11/ 356 (3.1) |
| Potassium (mmol/L) | Total | 3/1076 (0.3) | 2/1071 (0.2) | 1/ 356 (0.3) |
|  | < 3 | 2/1076 (0.2) | 1/1071 (0.1) | 0/ 356 (0) |
|  | > 6 | 1/1076 (0.1) | 1/1071 (0.1) | 1/ 356 (0.3) |
| Sodium (mmol/L) | Total | 0/1076 (0) | 0/1071 (0) | 0/ 356 (0) |
|  | < 125 | 0/1076 (0) | 0/1071 (0) | 0/ 356 (0) |
|  | > 160 | 0/1076 (0) | 0/1071 (0) | 0/ 356 (0) |
| Total Bilirubin (μmol/L) | Total | 2/1075 (0.2) | 3/1070 (0.3) | 2/ 356 (0.6) |
|  | > 34.2 | 2/1075 (0.2) | 3/1070 (0.3) | 2/ 356 (0.6) |

m, number of patients with a post-baseline value for the specified category; n, Number of patients meeting the criterion, i.e. who had a newly occurring clinically notable value or had a worsening of a value during treatment which was already notable at baseline; N, Total number of patients in the treatment group in this analysis set; ULN, upper limit of normal. SPIRIT baseline is used as the baseline value for all patients. For patients with a missing value at baseline, any post-baseline notable value is considered as newly occurring.

### Table S11. Number (%) of patients with newly occurring or worsening clinically notable on-treatment vital signs values at any time post-baseline using SPIRIT baseline

| **Variable**  **Abnormal category** | **Fevipiprant**  **150 mg**  **N=1092**  **n/m (%)** | **Fevipiprant**  **450 mg**  **N=1084**  **n/m (%)** | **Placebo**  **N=361**  **n/m (%)** |
| --- | --- | --- | --- |
| **Sitting Pulse Rate (bpm)** | | | |
| Total | 3/1083 (0.3) | 3/1074 (0.3) | 1/ 358 (0.3) |
| < 40 | 0/1083 (0) | 0/1074 (0) | 0/ 358 (0) |
| > 130 | 1/1083 (0.1) | 0/1074 (0) | 0/ 358 (0) |
| ≤ 50 and decrease from baseline by ≥ 15 | 2/1083 (0.2) | 2/1074 (0.2) | 1/ 358 (0.3) |
| ≥ 120 and increase from baseline by ≥ 15 | 1/1083 (0.1) | 1/1074 (0.1) | 0/ 358 (0) |
| **Sitting Systolic B.P. (mmHg)** | | | |
| Total | 8/1083 (0.7) | 11/1074 (1.0) | 2/ 358 (0.6) |
| < 75 | 0/1083 (0) | 0/1074 (0) | 0/ 358 (0) |
| > 200 | 0/1083 (0) | 0/1074 (0) | 0/ 358 (0) |
| ≤ 90 and decrease from baseline by ≥ 20 | 2/1083 (0.2) | 9/1074 (0.8) | 2/ 358 (0.6) |
| ≥ 180 and increase from baseline by ≥ 20 | 6/1083 (0.6) | 2/1074 (0.2) | 0/ 358 |
| **Sitting Diastolic B.P. (mmHg)** | | | |
| Total | 14/1083 (1.3) | 14/1074 (1.3) | 5/ 358 (1.4) |
| < 40 | 0/1083 (0) | 0/1074 (0) | 0/ 358 (0) |
| > 115 | 3/1083 (0.3) | 0/1074 (0) | 2/ 358 (0.6) |
| ≤ 50 and decrease from baseline by ≥ 15 | 4/1083 (0.4) | 8/1074 (0.7) | 3/ 358 (0.8) |
| ≥ 105 and increase from baseline by ≥ 15 | 10/1083 (0.9) | 6/1074 (0.6) | 2/ 358 (0.6) |
| **Weight (kg)** | | | |
| Total | 84/ 688 (12.2) | 83/ 648 (12.8) | 21/ 218 (9.6) |
| Decrease ≥ 7% from baseline | 35/ 688 (5.1) | 39/ 648 (6.0) | 11/ 218 (5.0) |
| Increase ≥ 7% from baseline | 49/ 688 (7.1) | 44/ 648 (6.8) | 10/ 218 (4.6) |

m, number of patients with a post-baseline value for the specified parameter; n, number of patients meeting the criterion, i.e. who had a newly occurring clinically notable value or had a worsening of a value during treatment which was already notable at baseline, N, Total number of patients in the treatment group in this analysis set. For patients with a missing value at baseline, any post-baseline notable value is considered as newly occurring. SPIRIT baseline is used as the baseline value for all patients.

### Table S12. Number (%) of patients with newly occurring or worsening clinically relevant on-treatment Fridericia's QTc values and increases from baseline at any time post-baseline using SPIRIT baseline

|  | **Fevipiprant**  **150 mg**  **N=1092**  **n/m (%)** | **Fevipiprant**  **450 mg**  **N=1084**  **n/m (%)** | **Placebo**  **N=361**  **n/m (%)** |
| --- | --- | --- | --- |
| **Variables QTcF (msec)** | | | |
| ≤ 450 for males | 6/ 428 (1.4) | 8/ 411 (1.9) | 3/ 132 (2.3) |
| ≤ 460 for females | 12/ 653 (1.8) | 11/ 663 (1.7) | 2/ 227 (0.9) |
| > 500 for males and females | 0/1081 (0) | 1/1074 (0.1) | 0/ 359 (0) |
| **Change from baseline in QTcF (msec)** |  |  |  |
| 30 - 60 | 66/1081 (6.1) | 52/1074 (4.8) | 21/ 359 (5.8) |
| > 60 | 2/1081 (0.2) | 1/1074 (0.1) | 0/ 359 (0) |

m, number of patients with a post-baseline value for the specified category; n, number of patients meeting the criterion, i.e. who had a newly occurring clinically notable value or had a worsening of a value during treatment which was already notable at baseline; N, total number of patients in the treatment group in this analysis set; QTc, corrected QT interval.; QTcF, Fridericia’s-corrected QTc interval. QTc categories are not mutually exclusive. SPIRIT baseline is used as the baseline value for all patients. For patients with a missing value at baseline, any post-baseline notable value is considered as newly occurring.

## D. Additional exploratory analysis for efficacy results

### Table S13. On-treatment analysis of rate of moderate-to-severe asthma exacerbations during the treatment period using negative binomial regression by population for patients rolled-over from the previous Phase 3 study

| **Treatment** | **Annualised rate (95% CI)** | **Comparison** | Rate  ratio | (95% CI) |
| --- | --- | --- | --- | --- |
| **Overall population** | | | | |
| **Fevipiprant 150 mg (n=442)** | 0.4 (0.4, 0.5) | Fevipiprant 150 mg / Placebo | 0.49 | (0.35, 0.69) |
| **Fevipiprant 450 mg (n=437)** | 0.4 (0.4, 0.5) | Fevipiprant 450 mg / Placebo | 0.48 | (0.34, 0.68) |
|  |  | Fevipiprant 450 mg / Fevipiprant 150 mg | 0.98 | (0.72, 1.32) |
| **Placebo (n=143)** | 0.9 (0.7, 1.2) |  |  |  |
| **Patients with blood eosinophil count ≥ 250 cells/µL** | | | | |
| **Fevipiprant 150 mg (n=302)** | 0.4 (0.3, 0.6) | Fevipiprant 150 mg / Placebo | 0.51 | (0.34, 0.77) |
| **Fevipiprant 450 mg (n=297)** | 0.5 (0.4, 0.6) | Fevipiprant 450 mg / Placebo | 0.58 | (0.38, 0.90) |
|  |  | Fevipiprant 450 mg / Fevipiprant 150 mg | 1.14 | (0.78, 1.65) |
| **Placebo (n=93)** | 0.9 (0.6, 1.2) |  |  |  |

Negative binomial regression model: log (exacerbation rate) = randomization stratum (fevipiprant 150mg in LUSTER-1/LUSTER-2, fevipiprant 450 mg in LUSTER-1/LUSTER-2, Placebo in LUSTER-1/LUSTER-2)+ treatment+ region + the natural logarithm of the number of asthma exacerbations in the 12 months prior to screening that required treatment per protocol (this is the 12 months prior to joining the previous study), and for overall population, plus blood eosinophil count at screening (≥250 cells/ul, <250 cells/ul); The log (duration of follow-up in years) is used as an off-set variable. A rate ratio <1 favors treatment group in the numerator of the ratio. Blood eosinophil count at screening visit of prior study is considered for rollover patients.

### Table S14. On-treatment analysis of rate of moderate-to-severe asthma exacerbations during the total treatment period using negative binomial regression by population for new patients

| **Treatment** | **Annualised rate (95% CI)** | **Comparison** | Rate  ratio | (95% CI) |
| --- | --- | --- | --- | --- |
| **Overall population** | | | | |
| **Fevipiprant 150 mg (n=308)** | 0.3 (0.2, 0.4) | Fevipiprant 150 mg / Placebo | 1.02 | (0.58, 1.81) |
| **Fevipiprant 450 mg (n=310)** | 0.3 (0.2, 0.4) | Fevipiprant 450 mg / Placebo | 0.95 | (0.53, 1.68) |
|  |  | Fevipiprant 450 mg / Fevipiprant 150 mg | 0.93 | (0.63, 1.37) |
| **Placebo (n=103)** | 0.3 (0.2, 0.5) |  |  |  |
| **Patients with blood eosinophil count ≥ 250 cells/µL** | | | | |
| **Fevipiprant 150 mg (n=157)** | 0.4 (0.3, 0.6) | Fevipiprant 150 mg / Placebo | 1.17 | (0.55, 2.46) |
| **Fevipiprant 450 mg (n=164)** | 0.4 (0.3, 0.5) | Fevipiprant 450 mg / Placebo | 1.07 | (0.50, 2.25) |
|  |  | Fevipiprant 450 mg / Fevipiprant 150 mg | 0.91 | (0.56, 1.49) |
| **Placebo (n=52)** | 0.3 (0.2, 0.7) |  |  |  |

Negative binomial regression model: log (exacerbation rate) = treatment + region+ the natural logarithm of the number of asthma exacerbations in the 12 months prior to screening that required treatment per protocol, and for overall population, plus blood eosinophil count at screening (≥ 250 cells/ul, <250 cells/ul). The log (duration of follow-up in years) is used as an off-set variable. A rate ratio <1 favors treatment group in the numerator of the ratio.

### Table S15. On-treatment analysis of rate of moderate-to-severe asthma exacerbations during the treatment period using negative binomial regression (continued therapy vs. withdrawing or changing the dose of fevipiprant) for patients who completed a prior Phase 3 study

| **Treatment in LUSTER**  **Treatment in SPIRIT** | **Annualised rate (95% CI)** | **Comparison** | **Rate**  **ratio** | **(95% CI)** |
| --- | --- | --- | --- | --- |
| **Fevipiprant 450 mg in LUSTER-1/LUSTER-2** | | | | |
| **Continued (n=239)** | 0.4 (0.3, 0.6) | Continued fevipiprant 450 mg / withdrawn | 0.43 | (0.24, 0.76) |
| **Lowered dose (n=47)** | 0.4 (0.2, 0.8) | Lowered fevipiprant dose/ withdrawn | 0.40 | (0.18, 0.89) |
| **Withdrawn (n=45)** | 0.9 (0.5, 1.7) | Continued fevipiprant 450 mg / Lowered fevipiprant dose | 1.09 | (0.54, 2.19) |
| **Fevipiprant 150 mg in LUSTER-1/LUSTER-2** | | | | |
| **Continued (n=249)** | 0.5 (0.4, 0.7) | Continued fevipiprant 150 mg / withdrawn | 0.44 | (0.26, 0.74) |
| **Increased dose (n=51)** | 0.5 (0.2, 0.8) | Increased fevipiprant dose/ withdrawn | 0.40 | (0.19, 0.82) |
| **Withdrawn (n=50)** | 1.2 (0.7, 1.9) | Increased fevipiprant dose/ continued fevipiprant 150 mg | 0.90 | (0.49, 1.65) |
| **Placebo in LUSTER-1/LUSTER-2** | | | | |
| **Fevipiprant 450 mg (n=147)** | 0.4 (0.3, 0.7) | Changed to fevipiprant 450 mg /continued placebo | 0.66 | (0.38, 1.14) |
| **Fevipiprant 150 mg (n=146)** | 0.5 (0.3, 0.7) | Changed to fevipiprant 150 mg /continued placebo | 0.69 | (0.40, 1.19) |
| **Continued placebo (n=48)** | 0.7 (0.4, 1.2) | Changed to fevipiprant 450 mg / changed to fevipiprant 150 mg | 0.96 | (0.63, 1.47) |

Negative binomial regression model for each subgroup: log (exacerbation rate) = treatment + region + blood eosinophil count at screening (≥ 250 cells/ul, <250 cells/ul) + the natural logarithm of the number of asthma exacerbations that required treatment per protocol in the 12 months prior to the screening of prior study.

The natural logarithm of the duration of on-treatment follow-up in years is used as an offset variable. A rate ratio <1 favors treatment group in the numerator of the ratio.

### Table S16. Analysis of on-treatment asthma exacerbation rate for patients who had at least one exacerbation each year in two consecutive years before joining Spirit (LUSTER rollover patients)

| **Treatment** | **N** | **Rate (95% CI)** | Comparison | Rate ratio | 95% CI |
| --- | --- | --- | --- | --- | --- |
| **Overall population** | | | | | |
| Fevipiprant 150 mg | 185 | 0.80 (0.65, 0.99) | fevipiprant 150 mg / placebo | 0.50 | (0.35, 0.71) |
| Fevipiprant 450 mg | 175 | 0.80 (0.64, 1.00) | fevipiprant 450 mg / placebo | 0.50 | (0.35, 0.72) |
| Placebo | 62 | 1.59 (1.19, 2.12) |  |  |  |
| **Patients with blood eosinophil count** ≥ **250 cells/µL** | | | | | |
| Fevipiprant 150 mg | 124 | 0.80 (0.62, 1.05) | fevipiprant 150 mg / placebo | 0.57 | (0.36, 0.89) |
| Fevipiprant 450 mg | 119 | 0.87 (0.66, 1.16) | fevipiprant 450 mg / placebo | 0.61 | (0.38, 1.00) |
| Placebo | 43 | 1.42 (0.97, 2.07) |  |  |  |

The model included randomisation stratum, treatment, region and the natural logarithm of the number of asthma exacerbations in the 24 months prior to screening. For the overall population, blood eosinophil count at screening (≥ 250 cells/µL, < 250 cells/µL) was also included as a covariate.

### Table S17. On-treatment mixed model repeated measures analysis of change from baseline in pre-dose FEV_1_ (L) at Week 52 by population

| **Week** | **Treatment** | **LS mean*, SE** | **Comparison** | **LS mean, SE** | **95% CI** |
| --- | --- | --- | --- | --- | --- |
| **Overall population** | | | | | |
| **Baseline** | **All** | 1.895 |  |  |  |
| **Week 52** | **Fevipiprant  150 mg** | 0.122, 0.0163 | fevipiprant 150 mg - placebo | 0.071, 0.0322 | (0.008, 0.134) |
|  | **Fevipiprant  450 mg** | 0.139, 0.0164 | fevipiprant 450 mg - placebo | 0.088, 0.0323 | (0.024, 0.151) |
|  |  |  | fevipiprant 450 mg - fevipiprant 150 mg | 0.017, 0.0237 | (-0.030, 0.063) |
|  | **Placebo** | 0.051, 0.0278 |  |  |  |
| **Patients with blood eosinophil count ≥ 250 cells/µL** | | | | | |
| **Baseline** | **All** | 1.864 |  |  |  |
| **Week 52** | **Fevipiprant 150 mg** | 0.142,  0.0224 | fevipiprant 150 mg - Placebo | 0.107, 0.0451 | (0.018, 0.195) |
|  | **Fevipiprant 450 mg** | 0.185, 0.0223 | fevipiprant 450 mg - Placebo | 0.150, 0.0452 | (0.061, 0.238) |
|  |  |  | fevipiprant 450 mg - fevipiprant 150 mg | 0.043, 0.0325 | (-0.021, 0.107) |
|  | **Placebo** | 0.035, 0.0392 |  |  |  |

CI, confidence interval; LS Mean, least squares mean; MMRM, mixed model repeated measures; SE, standard error. For patients who completed a prior Phase 3 study, prior study baseline is used as the baseline value. MMRM: change from baseline in pre-dose FEV_1_= treatment group+ visit+ randomization Stratum (fevipiprant 150 mg in LUSTER-1/LUSTER-2, fevipiprant 450 mg in LUSTER-1/LUSTER-2, Placebo in LUSTER-1/LUSTER-2, New patients)+ severity of asthma (GINA treatment steps 3, 4, and 5)+ region +baseline pre-dose FEV_1_+baseline pre-dose FEV_1_*visit+treatment*visit, and for overall population, plus blood eosinophil count at screening (≥ 250 cells/μL, <250 cells/μL).

*For baseline, LS mean represents absolute LS mean value across all treatment groups at baseline. For post-baseline visits, LS mean represents the LS mean of the change from baseline within the treatment group.

### Table S18. Summary of number of treatment emergent asthma exacerbation episodes requiring hospitalisations in all patients

|  | **Fevipiprant 150 mg (N=1092)** | **Fevipiprant 450 mg (N=1084)** | **Placebo**  **(N=361)** |
| --- | --- | --- | --- |
| **Number of asthma exacerbations per patient** | | | |
| **Mean (SD)** | 0 (0.29) | 0 (0.18) | 0.1 (0.31) |
| **Number of asthma exacerbations per patient (n [%])** | | | |
| **0** | 1062 (97.3) | 1068 (98.5) | 348 (96.4) |
| **1** | 23 (2.1) | 13 (1.2) | 7 (1.9) |
| **2** | 3 (0.3) | 2 (0.2) | 5 (1.4) |
| **3** | 2 (0.2) | 0 (0) | 1 (0.3) |
| **≥4** | 2 (0.2) | 1 (0.1) | 0 (0) |
| **Duration of asthma exacerbations on treatment per patient (days)** | | | |
| **n** | 30 | 16 | 13 |
| **Mean (SD)** | 13.8 (9.23) | 11.8 (8.38) | 12.3 (8.61) |
| **Number of years on treatment per patient** | | | |
| **Mean (SD)** | 1.0 (0.55) | 1.0 (0.55) | 1.0 (0.56) |
| **Proportion of patients with exacerbations on treatment** | | | |
| **%** | 2.7 | 1.5 | 3.6 |
| **Total number of exacerbations on treatment** | 44 | 21 | 20 |
| **Total number of years on treatment** | 1100.80 | 1080.17 | 364.15 |
| **Rate of exacerbations per person year** | 0.04 | 0.02 | 0.05 |
| **Asthma exacerbations leading to intubation, n (%)** | 0 (0) | 0 (0) | 1 (0.3) |

Only asthma exacerbations requiring hospitalisations are considered in the table. Each record of asthma exacerbation episode is considered as an adverse event regardless of the interval between two episodes, i.e., no collapsing of multiple records, which is different from the collapsing approach used in efficacy analysis. Rate of exacerbations per person year = total number of exacerbations / total number of treatment years. Total number of treatment years = sum of exposure to study drug expressed in years (days / 365.25).

## E. Inclusion criteria

### Patients completing a prior Phase 3 study of fevipiprant:

Patients eligible for inclusion in this study must have fulfilled all of the following criteria:

1a. Informed consent and assent (if applicable) obtained at Visit 1 or within 14 days

of Visit 1. Informed consent and assent (if applicable) obtained before any study

assessment was performed.

2a. Completion of the treatment period (on blinded study drug) of a prior Phase 3 study of

Fevipiprant (i.e., did not discontinue blinded study treatment prematurely).

3a. Patient could safely continue into the study as judged by the investigator.

### Patients who have not previously participated in a study of fevipiprant:

Patients eligible for inclusion in this study must have fulfilled all of the following criteria:

1b. Informed consent and assent (if applicable) obtained at Visit 1 or within 14 days

of Visit 1. Informed consent and assent (if applicable) obtained before any study

assessment was performed.

2b. Male and female patients at a minimum age of 12 years (or higher minimum age limit as

allowed by health authority and/or ethics committee/institutional review board (IRB) approvals).

3b. Patients must have had a diagnosis of asthma (according to GINA 2016) for a period of at least 24 months prior to screening visit (Visit 1).

4b. Patients were treated with GINA steps 4 or 5 standard-of-care (SoC) asthma therapy for at least 3 months prior to Visit 1. The doses must have been stable for at least 4 weeks prior to Visit 1.

5b. Demonstration of inadequate control of asthma based on an ACQ score ≥1.5 at Visit 1.

6b. For patients aged ≥18 years, FEV_1_ of ≤85% of the predicted normal value for the patient, after withholding bronchodilators at Visit 1. For patients aged 12 to <18 years, FEV_1_ of ≤90% of the predicted normal value for the patient, after withholding bronchodilators at

Visit 1.

NOTE: Withholding of bronchodilators prior to spirometry:

- Short-acting β2-agonists (SABAs) ≥ 6 hours;
- Long-acting β2-agonists (LABAs) given twice daily ≥ 12 hours;
- LABAs given once daily ≥ 24 hours;
- Fixed dose combinations of LABA and ICS given twice daily ≥ 12 hours;
- Fixed dose combinations of LABA and ICS given once daily ≥ 24 hours; and
- Long-acting muscarinic antagonists (LAMAs) ≥ 24 hours.

7b. A clinical diagnosis of asthma supported by at least one of the following:

- An increase of ≥12% and ≥200 ml in FEV_1_ approximately 10 to 15 minutes after

administration of 400 mcg of salbutamol/albuterol (or equivalent dose) prior to randomisation. Spacer devices are not permitted during reversibility testing. All patients must have performed a reversibility test at the Visit 1.

If reversibility was not demonstrated at Visit 1, the following historical information could be

used:

- Documented evidence of reversibility performed according to American Thoracic Society/European Respiratory Society (ATS/ERS) (ATS/ERS 2005) or country specific guidelines within the 2 years prior to Visit 1. Where a patient was assessed as eligible based on historical evidence of reversibility, a copy of the original printed spirometry report with relevant spirometry tracings must have been available as source documentation.
- Documented evidence of a positive airways hyper-responsiveness (AHR) test result within the 2 years prior to Visit 1, defined as a provoked fall in FEV_1_ of 20% by methacholine at ≤8 mg/ml (or histamine ≤10 mg/ml or acetylcholine <20 mg/mL) when not on ICS or ≤16 mg/ml (or histamine ≤20 mg/ml or acetylcholine <40 mg/mL) on ICS therapy performed according to ATS/ERS guidelines.

## F. Exclusion criteria

### Patients completing a prior Phase 3 study of fevipiprant:

Patients/subjects fulfilling any of the following criteria were not eligible for inclusion in this

study. No additional exclusions could be applied by the investigator, in order to ensure that the study population was representative of all eligible patients/subjects.

1a. Pregnant or nursing (lactating) women, where pregnancy is defined as the state of a female after conception and until the termination of gestation, confirmed by a positive hCG laboratory test.

2a. Women of child-bearing potential, defined as all women physiologically capable of becoming pregnant, unless they are using basic methods of contraception during dosing of study drug. Basic contraception methods include:

- Total abstinence (when this is in line with the preferred and usual lifestyle of the subject) if allowed as basic method of contraception by local regulations. Periodic abstinence (e.g., calendar, ovulation, symptothermal, post-ovulation methods) and withdrawal are not acceptable methods of contraception.
- Female sterilisation (have had surgical bilateral oophorectomy with or without hysterectomy) or tubal ligation at least six weeks before taking study treatment. In case of oophorectomy alone, only when the reproductive status of the woman has been confirmed by follow up hormone level assessment.
- Male sterilisation (at least 6 months prior to screening). For female subjects on the study, the vasectomised male partner must be the sole partner for that subject.
- Barrier methods of contraception: Condom or Occlusive cap (diaphragm or cervical/vault caps) if allowed as basic method of contraception by local regulations. For United Kingdom (UK): with spermicidal foam/gel/film/cream/ vaginal suppository.
- Use of oral, injected* or implanted* hormonal methods of contraception or other forms of hormonal contraception that have comparable efficacy (failure rate <1%), for example hormone vaginal ring or transdermal hormone contraception*.
- Placement of an intrauterine device (IUD) or intrauterine system (IUS).

*Not approved in Japan.

In case of use of oral contraception women must have been stable on the same pill for a

minimum of 3 months before taking study drug. Women are considered post-menopausal and not of child bearing potential if they have had 12 months of natural (spontaneous) amenorrhea with an appropriate clinical profile (e.g. age appropriate, history of vasomotor symptoms) or have had surgical bilateral oophorectomy (with or without hysterectomy) or tubal ligation at least six weeks previously. In the case of oophorectomy alone, only when the reproductive status of the woman had been confirmed by follow up hormone level assessment was she considered not of child bearing potential.

In case local regulations deviate from the contraception methods listed above, local

regulations apply and will be described in the ICF.

3a. Patients who did not complete the treatment period on blinded study drug of the prior Phase 3 study of fevipiprant they participated in.

4a. Inability to comply with all study requirements.

5a. Patients who experienced a serious and drug-related AE in the prior Phase 3 study of fevipiprant they participated in.

### Patients who have not previously participated in a study of fevipiprant:

Patients/subjects fulfilling any of the following criteria were not eligible for inclusion in this study. No additional exclusions could be applied by the investigator, in order to ensure that the study population was representative of all eligible patients/subjects.

1b. Use of other investigational drugs within 5 half-lives of enrolment, or within 30 days, whichever is longer.

2b. History of hypersensitivity to any of the study drugs or its excipients or to drugs of similar chemical classes to fevipiprant.

3b. History of lactose or milk sensitivity.

4b. Patients who had participated in another study of fevipiprant (i.e., the patient was randomised into another study of fevipiprant).

5b. Patients with a history or current diagnosis of ECG abnormalities indicating significant risk of safety for patients/subjects participating in the study such as: clinically significant cardiac arrhythmias, e.g., sustained ventricular tachycardia, and clinically significant second or third degree atrioventricular (AV) block without a pacemaker.

6b. History of familial long QT syndrome or known family history of Torsades de Pointes.

7b. Patients with a resting QTcF (Fridericia) ≥450 msec (male) or ≥460 msec (female) at Visit 1 or Visit 201.

8b. Use of any agent known to prolong the QT interval unless it could be permanently discontinued for the duration of the study.

9b. History of malignancy of any organ system (other than localised basal cell carcinoma

of the skin or in situ cervical cancer), treated or untreated, within the past 5 years,

regardless of whether there was evidence of local recurrence or metastases.

10b. Pregnant or nursing (lactating) women, where pregnancy was defined as the state

of a female after conception and until the termination of gestation, confirmed by a

positive hCG laboratory test.

11b. Women of child-bearing potential, defined as all women physiologically

capable of becoming pregnant, unless they are using basic methods of contraception

during dosing of study drug. Basic contraception methods include:

- Total abstinence (when this is in line with the preferred and usual lifestyle of the subject) if allowed as basic method of contraception by local regulations. Periodic abstinence (e.g., calendar, ovulation, symptothermal, post-ovulation methods) and withdrawal are not acceptable methods of contraception.
- Female sterilisation (have had surgical bilateral oophorectomy with or without hysterectomy) or tubal ligation at least six weeks before taking study treatment. In case of oophorectomy alone, only when the reproductive status of the woman has been confirmed by follow up hormone level assessment.
- Male sterilisation (at least 6 months prior to screening). For female subjects on the study, the vasectomised male partner must have been the sole partner for that subject.
- Barrier methods of contraception: Condom or Occlusive cap (diaphragm or cervical/vault caps) if allowed as basic method of contraception by local regulations. For UK: with spermicidal foam/gel/film/cream/ vaginal suppository.
- Use of oral, injected* or implanted* hormonal methods of contraception or other forms of hormonal contraception that have comparable efficacy (failure rate <1%), for example hormone vaginal ring or transdermal hormone contraception*.

*Not approved in Japan

- Placement of an intrauterine device (IUD) or intrauterine system (IUS). In case of use of oral contraception women must have been stable on the same pill for a minimum of 3 months before taking study drug.

Women are considered post-menopausal and not of child bearing potential if they have had 12 months of natural (spontaneous) amenorrhea with an appropriate clinical profile (e.g. age appropriate, history of vasomotor symptoms) or have had surgical bilateral oophorectomy (with or without hysterectomy) or tubal ligation at least six weeks ago. In the case of oophorectomy alone, only when the reproductive status of the woman has been confirmed by follow up hormone level assessment is she considered not of child bearing potential.

12b. Patients who had smoked or inhaled any substance other than asthma medications within the 6 month period prior to Visit 1, or who had a smoking history of greater than 10 pack years (Note:10 pack years = 1 pack /day x 10 yrs., or ½ pack/day x 20 yrs.).

13b. Patients who had an asthma exacerbation requiring systemic corticosteroids, hospitalisation, or emergency room visit within 6 weeks prior to Visit 1. If patients experienced an asthma exacerbation requiring systemic corticosteroids, hospitalisation or emergency room visit during screening, they could be rescreened once, ≥6 weeks after recovery from the exacerbation.

14b. Patients who had a respiratory tract infection or asthma worsening within 4 weeks of Visit 1. Patients who experienceed a respiratory tract infection or asthmaworsening during screening could be re-screened after 4 weeks after recovery from their respiratory tract infection or asthma worsening.

15b. Patients with any chronic condition of the respiratory tract which in the opinion of the investigator could interfere with study evaluation or optimal participation in the study.

16b. Patients with a history of chronic lung disease other than asthma, including (but not limited to) chronic obstructive pulmonary disease (as defined by Global Initiative for Chronic Obstructive Lung Disease (GOLD) standards),bronchiectasis, (non-clinically significant bronchiectasis may be allowed provided recent [within 3 months prior to Visit 1] CT scan proof was available), sarcoidosis, interstitial lung disease, cystic fibrosis, and tuberculosis.

17b. Patients with a history of conditions other than asthma, allergic rhinitis or sinusitis that could result in elevated eosinophils (e.g., hypereosinophilic syndromes, Churg-Strauss Syndrome, eosinophilic esophagitis). Patients with known parasitic infestation within 6 months prior to Visit 1 are also excluded.

18b. Patients with uncontrolled diabetes having an HbA_1c_ test result ≥8% at the Visit 1

laboratory test.

19b. Patients who had a clinically significant laboratory abnormality at the Visit 1 laboratory test including (but not limited to):

- Total white blood cell count <2500 cells/μL
- AST or ALT>2.0 X ULN or total bilirubin >1.3 X ULN
- Estimated Glomerular Filtration Rate (eGFR) by the Modification of Diet in Renal Disease (MDRD) equation or Bedside Schwartz equation <55 mL/minute/1.73 m2

20b. Patients who in the judgment of the investigator have a clinically significant condition such as (but not limited to) unstable ischemic heart disease, New York Heart Association (NYHA) Class III/IV left ventricular failure, arrhythmia,uncontrolled hypertension, cerebrovascular disease, neurodegenerative diseases, or other neurological disease, uncontrolled hypo- and hyperthyroidism and other autoimmune diseases, hypokalemia, hyperadrenergic state, or ophthalmologic disorder or patients with a medical condition that might compromise patient safety or compliance, interfere with evaluation, or preclude completion of the study.

21b. Patients with a history of myocardial infarction within 12 months of Visit 1.

22b. Patients with serious co-morbidities including, but not limited to, neurodegenerative diseases, rheumatoid arthritis and other autoimmune diseases.

23b. Patients with a history of alcohol or drug abuse within 12 months prior to Visit 1.

24b. Patients with a weight <30 kg.

25b. Patients aged 12 to <18 years below the 3rd percentile for weight by age (based on local growth charts or the United States Center for Disease Control growth charts, if local growth charts are not available) (Center for Disease Control and Prevention

2000).

26b. Patients receiving any medications in the classes listed in **Table S19** were excluded unless they met the criteria as specified in **Table S19**.

27b. Patients receiving medications in the classes listed in **Table S20** were excluded unless the medication had been stabilised for the specified period and the stated conditions had been met.

28b. Patients who started immunotherapy or desensitisation for allergies, within 3 months prior to Visit 1, or where the maintenance dose was expected to change during the study.

29b. Inability to comply with all study requirements.

30b. Patients with any medical or psychological condition that, in the investigators opinion, rendered the patient unable to understand the nature, scope, and possible consequences of the study.

31b. Patients with a history of being unable to swallow tablets.

32b. Patients who had received methotrexate, oral gold, troleandomycin, cyclosporine, azathioprine or any experimental anti-inflammatory therapies within 6 months of

Visit 1.

33b. Patients with regular use of oral or systemic corticosteroids for diseases other than asthma within the 12 months or any intra-articular or short-acting, intramuscular corticosteroid within 1 month or intramuscular, long acting depot corticosteroids within 3 months of Visit 1.

34b. Patients who have a history of or current treatment for hepatic disease including but not limited to acute or chronic hepatitis, cirrhosis or hepatic failure.

35b. Patients with a history of immunodeficiency disease or hepatitis B or hepatitis C.

36b. Patients on any statin therapy with a CK level >2 X ULN at Visit 1.

37b. Patients on >20 mg of simvastatin, > 40 mg of atorvastatin, >40 mg of pravastatin,

Or >2 mg of pitavastatin. Statin doses less than or equal to these doses as well as other statins were permitted during the study.

38b. Patients on rifampin, probenecid, ritonavir and valproic acid (i.e., medications blocking several pathways important for the elimination of fevipiprant [broad range UGT inhibition and/or inhibition of OAT3, OATP1B3, MXR and P-gp]).

39b. Patients who had received biologic therapy for the treatment of asthma within 5 months of Visit 1.

40b. No person directly associated with the administration of the study was allowed to participate as a study subject.

41b. No family member of the investigational study staff was allowed to participate in this study.

### Table S19. Prohibited medications

| **Class of medication** | **Minimum cessation prior to Visit 1** |
| --- | --- |
| Other investigational drugs | 30 days or 5 half-lives, whichever is longer |
| Live attenuated vaccine | 30 days |
| Other CRTH2 antagonists (e.g., ramatroban) | 7 days or 5 half-lives whichever is longer |
| Short-acting anticholinergics | 8 hours |
| Fixed combinations of SABAs and short-acting anticholinergics | 8 hours |
| Simvastatin >20 mg, atorvastatin >40 mg,  pravastatin >40 mg, or pitavastatin >2 mg  total daily dose | 7 days |
| Rifampin, probenecid, ritonavir and valproic  acid (i.e. medications blocking several  pathways important for the elimination of  fevipiprant (broad range UGT inhibition and/or inhibition of OAT3, OATP1B3, MXR and Pgp)). | 7 days |
| Methotrexate, gold salts, cyclosporine,  troleandomycin, azathioprine, other  immunomodulator drugs or  immunomodulatory monoclonal antibodies for  the treatment of conditions other than asthma | 6 months |

MXR, Multixenobiotic resistance; OAT, Organic Anion Transporters; P-gp, P-glycoprotein; UGT, UDP-glucuronosyltransferase

### Table S20. Medications allowed under certain conditions

| **Class of medication** | **Condition** |
| --- | --- |
| Inhaled corticosteroids (ICS) Medium-dose or High-dose ICS*. | Used for at least 3 months prior to Visit 1 and  stable for at least 4 weeks prior to Visit 1. |
| Long-acting inhaled β-2 agonists (LABAs) | Recommended doses and dosage regimens.  Used for at least 3 months prior to Visit 1 and  stable for at least 4 weeks prior to Visit 1.  Must be taken with an ICS. |
| Fixed dose combinations of ICS and LABA  (FDC) | Recommended doses and dosage regimens.  Used for at least 3 months prior to Visit 1 and  stable for at least 4 weeks prior to Visit 1. |
| Leukotriene receptor antagonists (LTRAs) | Recommended doses and dosage regimens.  Used for at least 3 months prior to Visit 1 and  stable for at least 4 weeks prior to Visit 1 |
| Theophylline | Recommended doses and dosage regimens  Used for at least 3 months prior to Visit 1 and  stable for at least 4 weeks prior to Visit 1 |
| Long-acting muscarinic antagonists  (LAMAs) | Recommended doses and dosage regimens  Used for at least 3 months prior to Visit 1 and  stable for at least 4 weeks prior to Visit 1. |
| Mast cell stabilizers (e.g., cromoglycate,  nedocromil, ketotifen) | Recommended doses and dosage regimens  Used for at least 3 months prior to Visit 1 and  stable for at least 4 weeks prior to Visit 1. |
| Maintenance oral corticosteroids for  treatment of asthma | Used for at least 3 months prior to Visit 1 and  stable for at least 4 weeks prior to Visit 1.  Note: Short bursts of rescue systemic  corticosteroids are allowed for treatment of asthma exacerbations, as clinically indicated. |
| .  Monoclonal antibodies for the treatment of  asthma | Recommended doses and dosage regimens  May be added as SoC asthma therapy during  study.  Must not have been administered less than 5  months prior to Visit 1. |
| SABAs | Rescue medication to be taken as needed. |
| Maintenance immunotherapy for allergies | Stable dose for at least 3 months prior to Visit 1  and the dose remains stable throughout the study. |
| Inactivated influenza vaccine,  pneumococcal vaccination or any other  inactivated vaccine | Not administered within 48 hours prior to a study  visit. |
| .  Topical corticosteroids for treatment of  eczema/ atopic dermatitis | Recommended doses and dosage regimens. |
| Antihistamines (e.g., loratadine, cetirzine) | Recommended doses and dosage regimens |
| Nasal anticholinergics | Treatment regimen has been stable for at least 1  month prior to Visit 1.  In the case of as needed use, providing an  established pattern of use has been documented. |
| Nasal corticosteroids |  |
| Nasal or ophthalmological preparations of  nedocromil |  |
| Nasal, ophthalmological, or oral preparations of antihistamines |  |

* Not applicable for patients entering this study from a prior Phase 3 study in which they were receiving low-dose ICS.

## G. Treatment Period 1 analysis

### Table S21. Time-to-first treatment-emergent adverse event, serious adverse event and adverse event leading to study treatment discontinuation – treatment period 1

| **Treatment** | **n (%)** | **Comparison** | **Hazard ratio** | **95% CI** |
| --- | --- | --- | --- | --- |
| **Time to 1^st^ TEAE in 52 weeks** | | | | |
| **Fevipiprant 150 mg (m=1081)** | 675  (62.4) | Fevipiprant 150 mg / Placebo | 0.86 | (0.74, 1.00) |
| **Fevipiprant 450 mg (m=1077)** | 654  (60.7) | Fevipiprant 450 mg / Placebo | 0.84 | (0.72, 0.97) |
| **Placebo (m=359)** | 237  (66.0) | Fevipiprant 450 mg / Fevipiprant 150 mg | 0.97 | (0.87, 1.09) |
|  |  |  |  |  |
| **Time to 1st treatment emergent SAE in 52 weeks** | | | | |
| **Fevipiprant 150 mg (m=1081)** | 73  (6.8) | Fevipiprant 150 mg / Placebo | 0.78 | (0.51, 1.22) |
| **Fevipiprant 450 mg (m=1077)** | 53  (4.9) | Fevipiprant 450 mg / Placebo | 0.61 | (0.39, 0.97) |
| **Placebo (m=359)** | 29  (8.1) | Fevipiprant 450 mg / Fevipiprant 150 mg | 0.78 | (0.54, 1.14) |
|  |  |  |  |  |
| **Time to 1st TEAE leading to treatment discontinuation in 52 weeks** | | | | |
| **Fevipiprant 150 mg (m=1081)** | 26  (2.4) | Fevipiprant 150 mg / Placebo | 0.98 | (0.47, 2.23) |
| **Fevipiprant 450 mg (m=1077)** | 33  (3.1) | Fevipiprant 450 mg / Placebo | 1.18 | (0.59, 2.64) |
| **Placebo (m=359)** | 9  (2.5) | Fevipiprant 450 mg / Fevipiprant 150 mg | 1.20 | (0.70, 2.10) |

AE, adverse event, m, total number of patients included in the analysis; n, number of patients with at least one event; SAE, serious adverse event, TEAE, treatment emergent adverse event. The Cox regression model = treatment group+ severity of asthma (GINA treatment steps 3, 4 and 5)+ region as fixed class effects, stratified by randomization stratum (fevipiprant 150 mg qd in LUSTER-1/LUSTER-2, fevipiprant 450 mg qd in LUSTER-1/LUSTER-2, Placebo in LUSTER-1/LUSTER-2, fevipiprant 150 mg qd in ZEAL-1/ZEAL-2, Placebo in ZEAL-1/ZEAL-2, New patients). Patients without the event of interest will be censored at the minimum out of the dates of last medication intake in treatment period 1 + 30 days, final visit date in treatment period 1, and date of death. A hazard ratio < 1 favors the treatment group in the numerator of the ratio.

### Table S22. Summary of number of treatment emergent asthma exacerbation episodes requiring hospitalisations in all patients – treatment period 1

|  | **Fevipiprant 150 mg (N=1092)** | **Fevipiprant 450 mg (N=1084)** | **Placebo**  **(N=361)** |
| --- | --- | --- | --- |
| **Number of asthma exacerbations per patient** | | | |
| **Mean (SD)** | 0 (0.26) | 0 (0.14) | 0 (0.25) |
| **Number of asthma exacerbations per patient (n [%])** | | | |
| **0** | 1067 (97.7) | 1070 (98.7) | 349 (96.7) |
| **1** | 21 (1.9) | 12 (1.1) | 8 (2.2) |
| **2** | 1 (0.1) | 2 (0.2) | 4 (1.1) |
| **3** | 1 (0.1) | 0 (0) | 0 (0) |
| **≥4** | 2 (0.2) | 0 (0) | 0 (0) |
| **Duration of asthma exacerbations on treatment per patient (days)** | | | |
| **n** | 25 | 14 | 12 |
| **Mean (SD)** | 18.4 (13.68) | 13.8 (8.92) | 14.7 (10.37) |
| **Number of years on treatment per patient** | | | |
| **Mean (SD)** | 0.8 (0.27) | 0.8 (0.28) | 0.8 (0.27) |
| **Proportion of patients with exacerbations on treatment** | | | |
| **%** | 2.3 | 1.3 | 3.3 |
| **Total number of exacerbations on treatment** | 35 | 16 | 16 |
| **Total number of years on treatment** | 860.72 | 843.3 | 284.64 |
| **Rate of exacerbations per person year** | 0.04 | 0.02 | 0.06 |
| **Asthma exacerbations leading to intubation, n(%)** | 0 (0) | 0 (0) | 1 (0.3) |

Only asthma exacerbations requiring hospitalisations are considered in the table. Each record of asthma exacerbation episode is considered as an adverse event regardless of the interval between two episodes, i.e., no collapsing of multiple records, which is different from the collapsing approach used in efficacy analysis. Rate of exacerbations per person year = total number of exacerbations / total number of treatment years. Total number of treatment years = sum of exposure to study drug expressed in years (days / 365.25).

There were no patient deaths due to due to a treatment emergent asthma exacerbation in treatment period 1.

### Table S23. Analysis of the incidence rate of treatment emergent adverse events by primary system organ class using logistic regression - treatment period 1

| **Primary system organ class** | **Treatment** | **n (%)** | **Comparison** | **Odds ratio** | **(95% CI)** |
| --- | --- | --- | --- | --- | --- |
| Any SOC | Fevipiprant 450mg (N=1077) | 654 (60.7) | Fevipiprant 450mg/ Placebo | 0.8 | 0.622,1.038 |
|  | Fevipiprant 150mg (N=1081) | 675 (62.4) | Fevipiprant 150mg/ Placebo | 0.81 | 0.626, 1.047 |
|  | Placebo (N=359) | 237 (66.0) | Fevipiprant 450mg/150mg | 0.99 | 0.821, 1.200 |
| Blood and lymphatic system disorders | Fevipiprant 450mg (N=1077) | 10 (0.9) | Fevipiprant 450mg/ Placebo | 0.69 | 0.259, 1.862 |
|  | Fevipiprant 150mg (N=1081) | 21 (1.9) | Fevipiprant 150mg/ Placebo | 1.13 | 0.460, 2.793 |
|  | Placebo (N=359) | 5 (1.4) | Fevipiprant 450mg/150mg | 0.61 | 0.291, 1.289 |
| Cardiac disorders | Fevipiprant 450mg (N=1077) | 26 (2.4) | Fevipiprant 450mg/ Placebo | 1.19 | 0.540, 2.643 |
|  | Fevipiprant 150mg (N=1081 | 11 (1.0) | Fevipiprant 150mg/Placebo | 0.48 | 0.197, 1.177 |
|  | Placebo (N=359) | 7 (1.9) | Fevipiprant 450mg/150mg | 2.48 | 1.214, 5.080 |
| Congenital, familial and genetic disorders | Fevipiprant 450mg (N=1077) | 3 (0.3) | Fevipiprant 450mg/ Placebo | 3.06 | 0.341, 27.420 |
|  | Fevipiprant 150mg (N=1081) | 1 (0.1) | Fevipiprant 150mg/Placebo | 0.85 | 0.079, 9.100 |
|  | Placebo (N=359) | 0 (0) | Fevipiprant 450mg/ 150mg | 3.6 | 0.825, 15.677 |
| Ear and labyrinth disorders | Fevipiprant 450mg (N=1077) | 9 (0.8) | Fevipiprant 450mg/ Placebo | 0.35 | 0.145, 0.858 |
|  | Fevipiprant 150mg (N=1081) | 16 (1.5) | Fevipiprant 150mg/ Placebo | 0.63 | 0.281, 1.401 |
|  | Placebo (N=359) | 8 (2.2) | Fevipiprant 450mg/150mg | 0.56 | 0.256, 1.233 |
| Endocrine disorders | Fevipiprant 450mg (N=1077) | 6 (0.6) | Fevipiprant 450mg/ Placebo | 3.19 | 0.321, 31.679 |
|  | Fevipiprant 150mg (N=1081) | 2 (0.2) | Fevipiprant 150mg/ Placebo | 2.12 | 0.191, 23.535 |
|  | Placebo (N=359) | 0 (0) | Fevipiprant 450mg/150mg | 1.50 | 0.449, 5.028 |
| Eye disorders | Fevipiprant 450mg (N=1077) | 11 (1.0) | Fevipiprant 450mg/ Placebo | 0.35 | 0.151, 0.796 |
|  | Fevipiprant 150mg (N=1081 | 13 (1.2) | Fevipiprant 150mg/Placebo | 0.45 | 0.202, 1.020 |
|  | Placebo (N=359) | 9 (2.5) | Fevipiprant 450mg/150mg | 0.76 | 0.347, 1.679 |
| Gastrointestinal disorders | Fevipiprant 450mg (N=1077) | 86 (8.0) | Fevipiprant 450mg/ Placebo | 0.84 | 0.548, 1.282 |
|  | Fevipiprant 150mg (N=1081) | 88 (8.1) | Fevipiprant 150mg/ Placebo | 0.9 | 0.590, 1.380 |
|  | Placebo (N=359) | 32 (8.9) | Fevipiprant 450mg/150mg | 0.93 | 0.671, 1.287 |
| General disorders and administration site conditions | Fevipiprant 450mg (N=1077) | 33 (3.1) | Fevipiprant 450mg/ Placebo | 1.5 | 0.681, 3.287 |
|  | Fevipiprant 150mg (N=1081) | 38 (3.5) | Fevipiprant 150mg/ Placebo | 1.61 | 0.737, 3.495 |
|  | Placebo (N=359) | 7 (1.9) | Fevipiprant 450mg/150mg | 0.93 | 0.571, 1.521 |
| Hepatobiliary disorders | Fevipiprant 450mg (N=1077) | 17 (1.6) | Fevipiprant 450mg/ Placebo | 0.78 | 0.344, 1.788 |
|  | Fevipiprant 150mg (N=1081 | 9 (0.8) | Fevipiprant 150mg/Placebo | 0.41 | 0.166, 1.032 |
|  | Placebo (N=359) | 7 (1.9) | Fevipiprant 450mg/150mg | 1.89 | 0.857, 4.185 |
| Immune system disorders | Fevipiprant 450mg (N=1077) | 10 (0.9) | Fevipiprant 450mg/ Placebo | 0.45 | 0.174, 1.140 |
|  | Fevipiprant 150mg (N=1081) | 11 (1.0) | Fevipiprant 150mg/Placebo | 0.61 | 0.241, 1.534 |
|  | Placebo (N=359) | 6 (1.7) | Fevipiprant 450mg/ 150mg | 0.73 | 0.310, 1.730 |
| Infections and infestations | Fevipiprant 450mg (N=1077) | 374 (34.7) | Fevipiprant 450mg/ Placebo | 0.78 | 0.607, 1.003 |
|  | Fevipiprant 150mg (N=1081) | 400 (37.0) | Fevipiprant 150mg/ Placebo | 0.83 | 0.648, 1.067 |
|  | Placebo (N=359) | 145 (40.4) | Fevipiprant 450mg/150mg | 0.94 | 0.775, 1.136 |
| Injury poisoning and procedural complications | Fevipiprant 450mg (N=1077) | 52 (4.8) | Fevipiprant 450mg/ Placebo | 0.72 | 0.438, 1.197 |
|  | Fevipiprant 150mg (N=1081) | 67 (6.2) | Fevipiprant 150mg/ Placebo | 0.93 | 0.573, 1.518 |
|  | Placebo (N=359) | 23 (6.4) | Fevipiprant 450mg/150mg | 0.78 | 0.526, 1.147 |
| Investigations | Fevipiprant 450mg (N=1077) | 85 (7.9) | Fevipiprant 450mg/ Placebo | 1.46 | 0.890, 2.410 |
|  | Fevipiprant 150mg (N=1081 | 79 (7.3) | Fevipiprant 150mg/Placebo | 1.31 | 0.791, 2.156 |
|  | Placebo (N=359) | 20 (5.6) | Fevipiprant 450mg/150mg | 1.12 | 0.800, 1.572 |
| Metabolism and nutrition disorders | Fevipiprant 450mg (N=1077) | 50 (4.6) | Fevipiprant 450mg/ Placebo | 0.75 | 0.444, 1.261 |
|  | Fevipiprant 150mg (N=1081) | 53 (4.9) | Fevipiprant 150mg/ Placebo | 0.81 | 0.483, 1.361 |
|  | Placebo (N=359) | 21 (5.8) | Fevipiprant 450mg/150mg | 0.92 | 0.608, 1.402 |
| Musculoskeletal and connective tissue disorders | Fevipiprant 450mg (N=1077) | 84 (7.8) | Fevipiprant 450mg/ Placebo | 1.16 | 0.729, 1.858 |
|  | Fevipiprant 150mg (N=1081) | 91 (8.4) | Fevipiprant 150mg/ Placebo | 1.23 | 0.771, 1.953 |
|  | Placebo (N=359) | 24 (6.7) | Fevipiprant 450mg/150mg | 0.95 | 0.683, 1.319 |
| Neoplasms benign, malignant and unspecified (incl cysts and polyps) | Fevipiprant 450mg (N=1077) | 9 (0.8) | Fevipiprant 450mg/ Placebo | 0.96 | 0.309, 3.010 |
|  | Fevipiprant 150mg (N=1081 | 13 (1.2) | Fevipiprant 150mg/Placebo | 1.2 | 0.402, 3.602 |
|  | Placebo (N=359) | 3 (0.8) | Fevipiprant 450mg/150mg | 0.8 | 0.356, 1.799 |
| Nervous system disorders | Fevipiprant 450mg (N=1077) | 73 (6.8) | Fevipiprant 450mg/ Placebo | 0.84 | 0.539, 1.319 |
|  | Fevipiprant 150mg (N=1081) | 67 (6.2) | Fevipiprant 150mg/Placebo | 0.72 | 0.456, 1.126 |
|  | Placebo (N=359) | 29 (8.1) | Fevipiprant 450mg/ 150mg | 1.18 | 0.819, 1.691 |
| Product issues | Fevipiprant 450mg (N=1077) | 0 (0) | Fevipiprant 450mg/ Placebo | 0.32 | 0.035, 3.019 |
|  | Fevipiprant 150mg (N=1081) | 1 (0.1) | Fevipiprant 150mg/ Placebo | 1.09 | 0.165, 7.133 |
|  | Placebo (N=359) | 0 (0) | Fevipiprant 450mg/150mg | 0.30 | 0.046, 1.913 |
| Psychiatric disorders | Fevipiprant 450mg (N=1077) | 15 (1.4) | Fevipiprant 450mg/ Placebo | 0.48 | 0.218, 1.058 |
|  | Fevipiprant 150mg (N=1081) | 18 (1.7) | Fevipiprant 150mg/ Placebo | 0.66 | 0.308, 1.431 |
|  | Placebo (N=359) | 9 (2.5) | Fevipiprant 450mg/150mg | 0.72 | 0.365, 1.435 |
| Renal and urinary disorders | Fevipiprant 450mg (N=1077) | 41 (3.8) | Fevipiprant 450mg/ Placebo | 1.76 | 0.842, 3.682 |
|  | Fevipiprant 150mg (N=1081 | 33 (3.1) | Fevipiprant 150mg/Placebo | 1.22 | 0.577, 2.586 |
|  | Placebo (N=359) | 8 (2.2) | Fevipiprant 450mg/150mg | 1.44 | 0.884, 2.347 |
| Reproductive system and breast disorders | Fevipiprant 450mg (N=1077) | 9 (0.8) | Fevipiprant 450mg/ Placebo | 0.73 | 0.254, 2.083 |
|  | Fevipiprant 150mg (N=1081) | 14 (1.3) | Fevipiprant 150mg/ Placebo | 1.04 | 0.384, 2.819 |
|  | Placebo (N=359) | 4 (1.1) | Fevipiprant 450mg/150mg | 0.70 | 0.313, 1.562 |
| Respiratory, thoracic and mediastinal disorders | Fevipiprant 450mg (N=1077) | 304 (28.2) | Fevipiprant 450mg/ Placebo | 0.66 | 0.507, 0.848 |
|  | Fevipiprant 150mg (N=1081) | 308 (28.5) | Fevipiprant 150mg/ Placebo | 0.61 | 0.473, 0.793 |
|  | Placebo (N=359) | 135 (37.6) | Fevipiprant 450mg/150mg | 1.07 | 0.873, 1.313 |
| Skin and subcutaneous tissue disorders | Fevipiprant 450mg (N=1077) | 39 (3.6) | Fevipiprant 450mg/ Placebo | 0.98 | 0.524, 1.839 |
|  | Fevipiprant 150mg (N=1081 | 38 (3.5) | Fevipiprant 150mg/Placebo | 0.91 | 0.483, 1.702 |
|  | Placebo (N=359) | 13 (3.6) | Fevipiprant 450mg/150mg | 1.08 | 0.673, 1.740 |
| Social circumstances | Fevipiprant 450mg (N=1077) | 1 (0.1) | Fevipiprant 450mg/ Placebo | 0.14 | 0.033, 0.598 |
|  | Fevipiprant 150mg (N=1081) | 1 (0.1) | Fevipiprant 150mg/Placebo | 0.14 | 0.034, 0.613 |
|  | Placebo (N=359) | 3 (0.8) | Fevipiprant 450mg/ 150mg | 0.98 | 0.173, 5.570 |
| Vascular disorders | Fevipiprant 450mg (N=1077) | 33 (3.1) | Fevipiprant 450mg/ Placebo | 1.21 | 0.592, 2.485 |
|  | Fevipiprant 150mg (N=1081) | 39 (3.6) | Fevipiprant 150mg/ Placebo | 1.37 | 0.678, 2.777 |
|  | Placebo (N=359) | 9 (2.5) | Fevipiprant 450mg/150mg | 0.88 | 0.545, 1.433 |

n, number of patients with at least one treatment emergent adverse event. Logistic regression model: logit (proportion) = randomization stratum (fevipiprant150 mg in LUSTER-1/LUSTER-2, fevipiprant 450 mg in LUSTER-1/LUSTER-2, Placebo in LUSTER-1/LUSTER-2, fevipiprant 150 mg in ZEAL-1/ZEAL-2, Placebo in ZEAL-1/ZEAL-2, New patients), treatment, severity of asthma (GINA steps 3, 4 and 5), region as fixed effects. An odds ratio < 1 favours the treatment group in the numerator of the ratio

## H. Additional statistical methods

A full description of the statistical analyses performed for each study is contained in the study statistical analysis plan (SAP). Additional material on randomisation and allocation not contained in the SAP is given below.

Stratified block randomisation was used. The randomization numbers were generated using the following procedure to ensure that treatment assignment was unbiased and concealed from patients and investigator staff. A patient randomization list was produced by the Interactive Response Technology (IRT) provider (Cenduit) using a validated system that automated the random assignment of patient numbers to randomization numbers. These randomization numbers were linked to the different treatment arms, which in turn were linked to medication numbers. A separate medication list was produced under the responsibility of Novartis Global Clinical Supplies (GCS) using a validated system that automated the random assignment of medication numbers to packs containing the investigational drug. The randomization scheme for patients was reviewed and approved by a member of the Novartis Randomization Office.

Patients were randomised in an approximate ratio of 3:3:1 to one of three treatments (QAW039 150 mg once daily, fevipiprant 450 mg once daily or placebo once daily). Randomised patients were stratified according to randomization stratum below.

- patients on fevipiprant 150 mg once daily treatment in the LUSTER-1 and LUSTER-2 studies,
- patients on fevipiprant 450 mg once daily treatment the LUSTER-1 and LUSTER-2 studies,
- patients on placebo in the LUSTER-1 and LUSTER-2 studies
- patients on fevipiprant 150 mg once daily treatment in the ZEAL-1 and ZEAL-2 studies,
- patients on placebo in the ZEAL-1 and ZEAL-2 studies,
- patients who have not previously participated in a study of fevipiprant,

Treatment randomization was stratified at the regional level (Europe, LaCan-Canada, China/AMAC, US+Canada, Japan). A block size of 7 was used.

## I. Participating investigators

### Table S24. List of participating investigators for the SPIRIT study

| **Country** | **Participating investigator** |
| --- | --- |
| Argentina | Ledit Ardusso, Horacio Budani, Alexis Cazaux, Ricardo del Olmo, Cristian Fazio, Marcelo Fernandez, Gabriel Gattolin, Maria Cecilia Hasner, Martin Maillo, Adriana Marcipar, Gustavo Marino, Jorge Maspero, Fernando Massola, Andrea Medina, Alicia Molina, Maria Otaola, Federico Promencio, Ramon A Rojas, Gisela Delgado Vizcarra, Luis Wehbe, Maricel Willigs Rolon, Anahi Yanez |
| Australia | Philip Bardin, Mark Hew, Dimitar Sajkov, AnneMarie Southcott |
| Austria | Marco Idzko, Gerhard Koeberl, Wolfgang Pohl |
| Belgium | Antoine Bolly, Maud Deschampheleire, Elke Hardeman, Sofie Maddens, Jean Benoit Martinot, Yves Mentens, Olivier Michel, Vincent Ninane |
| Brazil | Martti Antila, Daniela Blanco, Fabio Fernandes Morato Castro, Marina Lima, Andreia Pez, Marcelo Rabahi, Adalberto Sperb Rubin, Rafael Stelmach |
| Bulgaria | Dimo Dimov, Elena Docheva,Yavor Ivanov, Mariana Mandazhieva-Pepelanova |
| Canada | Syed Anees, Kenneth Chapman, Jaime Del Carpio, Delbert Dorscheid, Anthony DUrzo, Anne Ellis, Mark Fitzgerald, Remi Gagnon, Anil Gupta, Catherine Lemiere, Carmela Pepe |
| China | Ping Chen, Jing Li, Weimin Li, Jinming Liu, Xiaoxia Liu, Liping Peng, Changzheng Wang, Yan Xixin, Lan Yang, Li Zhao, Xiaoli Zhu |
| Colombia | Diego Fernando Acosta Hoyos, Diana Cano, Mauricio Duran, Francisco Orlando Serrano Vasquez |
| Czech Republic | Tomas Dvorak, Stanislav Holub, Daniela Kopecka, Tomas Rohovsky, Vladimir Zindr |
| Estonia | Rain Jogi, Pille Mukk |
| Finland | Auli Hakulinen, Jussi Karjalainen |
| France | Francois Xavier Blanc, Philippe Bonniaud, Arnaud Bourdin, Pascal Chanez, Frederic de Blay, Gilles Devouassoux, Gilles Garcia, Camille Taille |
| Germany | Karl Heinz Franz, Christian Gessner, Frank Kaessner, Claus Keller, Oliver Kornmann, Matthias Kruell, Anneliese Linnhoff, Hilke Maria Temme, Peter Rueckert, Thomas Schultz, Heiner Steffen |
| Greece | Elpiniki Georgatou Papageorgiou, Evangelia Kompoti, Ioannis Paraskevopoulos, Konstantinos Porpodis, Fotios Psarros, Ekaterini Syrigou, Eleftherios Zervas |
| Guatemala | Edgar Contreras, Onam Esau Espana, Xiomara Gamboa, Jeremias Guerra, Gerardo Martinez, Erick Yoc |
| Hungary | Beatrix Balint, Aranka Banvolgyi, Anna Bartha, Zoltan Bende, Marta Czompo, Edit Dulka, Nelli Kosztyu, Aniko Kurucz, Janos Mucsi, Marta Papp, Eva Radeczky, Judit Schlezak, Katalin Udud |
| India | Srikanth Krishnamurthy, Surendra Kumar, Vinit Niranjane, Amit Patel, Jagdish Kumar Rawat, Sundeep Salvi, Sharad Tikkiwal, Manoj Yadav |
| Israel | Yochai Adir, Amir Bar Shai, Lea Bentur, Neville Berkman, Gershon Fink, Gabriel Izbicki, Mordechai Kramer |
| Japan | Tomoyuki Fujisawa, Takao Fujisawa, Satoshi Fuke, Yasushi Fukushima, Yasuhiro Gon, Osamu Hataji, Yumiko Ide, Motoyasu Iikura, Nobuhisa Ishikawa, Uguri Kamiya, Takanori Kanematsu, Motokazu Kato, Yuji Kawarada, Norio Kihara, Masaharu Kinoshita, Takaharu Kizu, Makoto Kudo, Kentaro Machida, Seijiro Minamoto, Naoki Miyazawa, Nobuhiko Nagata, Kiyotaka Nakajima, Hiroyuki Nakamura, Norihiko Nakanishi, Yuji Nakatani, Akio Niimi, Naoki Nishimura, Hiroyuki Ohbayashi, Nobuharu Oshima, Hironori Sagara, Takefumi Saito, Osamu Sakamoto, Kiyoshi Sekiya, Atsushi Shibasaki, Jun Shinada, Tsuneyuki Takahashi, Shohei Takata, Satomi Takeuchi, Hiroshi Tanaka, Kazunori Tobino, Keiko Wakahara, Tsuyoshi Yamato, Makoto Yoshida, Masanori Yoshikawa |
| Latvia | Aurika Babjoniseva, Nadezda Kolosa, Lilita Mitrofanova |
| Lebanon | Zuheir Alameh, Juvelekian Georges, Carla Irany |
| Lithuania | Inga Davoliene, Giedre Grigiene, Kestutis Malakauskas, Vitalija Nausediene, Daina Susinskiene, Jolita Vebriene |
| Malaysia | Umadevi A Muthukumaru, Aishah Ibrahim, Ahmad Izuanuddin Ismail, Mat Zuki Mat Jaeb |
| Mexico | Jesus Diaz Castanon, Efrain Montano, Guillermo Montes, Socorro Orozco, Francisco Sanchez Llamas |
| Netherlands | Anneke ten Brinke, Marjo van de Ven |
| Peru | Ronal Gamarra, Alfredo Guerreros, Victor Oswaldo Lizarbe, Fernando Rodriguez, Danilo Joel Salazar Ore, Cesar Villaran |
| Philippines | Aileen David Wang, Marie Grace Dawn Isidro  Teresita De Guia, Dr Tabag, Jose Felipe G Hernandez, Araceli Maliwat, Camilo Roa Jr |
| Poland | Lucyna Dymek, Teresa Hofman, Danuta Madra-Rogacka, Zenon Siergiejko, Ewa Springer |
| Romania | Ioana Agache, Dana Sorina Alexandrescu, Liliana Duca, Milena Adina Man, Cristina Mihaela Ciobanu, Stefan Mihaicuta, Ioan Dorin Petrui, Doina Piriu, Angelica Savu, Ana Maria Trailescu, Voicu Tudorache |
| Russia | Alina Agafina, Galina Lvovna Ignatova, Veronika Kadochnikova, Yaroslava Khovaeva, Oksana Kurbacheva, Vladimir Martynenko, Sergey Mikhaylov, Vladimir Nosov, Ekaterina Orlova, Veronika Popova, Tamara Rubanik, Natalia Shaporova, Vasilii Trofimov, Olga Ukhanova, Maxim Vasiliev, Elena Mikhailovna Vishneva |
| Saudi Arabia | Amr Al-Banna, Hani Lababidi |
| Serbia | Ivan Cekerevac, Ivan Kopitovic, Gordana Kostic, Branislava Milenkovic, Predrag Minic, Ivana Stankovic |
| Singapore | Lim Hui Fang, Tunn Ren Tay |
| Slovakia (Slovak Republic) | Luboslava Frajtova, Svetlana Hadvabova, Daniela Hasicova, Ivan Hlinka, Slavomir Hrebenar, Peter Jurco, Denisa Kavkova, Helena Lescisinova ,Jan Plutinsky, Jana Zelnikova |
| Spain | Carmen Alvarez Sanchez, Irina Bobolea, Paloma Campo Mozo, Jose Echave-Sustaeta, Borja Garcia Cosio Piqueras, Juan Luis Garcia Rivero, Francisco Javier Gonzalez Barcala, Jose Maria Ignacio Garcia, Luis Perez de Llano, Juan Roldan Sanchez, David Romero, Jose Gregorio Soto, Arantza Vega Castro, Jose Luis Velasco, Elisabeth Vera Solsona |
| Switzerland | Joerg Leuppi, Alessia Rigglieti |
| Taiwan | Jeng Yuan Hsu |
| Tunisia | Sonia Toujani |
| Turkey | Serir Aktogu Ozkan, Bilun Gemicioglu, Ismail Hanta, Mehmet Karadag, Dilsad Mungan, Sibel Nayci, Mediha Ortakoylu, Insu Ylmaz |
| United Kingdom | Christopher Brightling, Shoaib Faruqi, Shuaib Nasser, Monica Nordstrom, Mitesh Patel, Dinesh Saralaya |
| United States | Kevin Ahlers, Akinyemi Ajayi, Ian Alexander, Vivek Awasty, Ian Baird, Alan Baptist, George Bensch, David Bernstein, David Bouda, Tracy Bridges, James Carswell IV, David M Cypcar, James Deangelo, Samuel Deleon, Dennis Dilley, David Elkayam, Faisal M Fakih, Gary Ferguson, Sarah Samimi Field, David Fitz-Patrick, Ramon Flores Rios, Linda Ford, Binu George, Glenn Giessel, Elliot Ginchansky, Shaila Gogate, Richard Gower, Fred Grynberg, Michael Hull, Joshua Jacobs, Mikell Jarratt, Devi Jhaveri, Edward Kerwin, Yasuko Kidokoro, Ryan Klein, Shahrukh Kureishy, Douglas Mapel, Thomas Minor, Lee Morrow, Mark Neustrom, David Nyanjom, David Pham, Warren Pleskow, Bruce Prenner, Sudhir Sekhsaria, Russell Settipane, Paul A Shapero PA, Sayantani Sindher, David Smith, Andrew Smith, Weily Soong, Roy St John, Barry Streit, James Sublett, Martha Tarpay, Ernest Thompson, Carl Thornblade, Swapnil Vaidya, Luke Webb, Pamela Zeitlin |
